# Bioinformatics and Next-Generation Data Analysis for Identification of Genes and Molecular Pathways Involved in Subjects with Diabetes and Obesity

**DOI:** 10.3390/medicina59020309

**Published:** 2023-02-07

**Authors:** Prashanth Ganekal, Basavaraj Vastrad, Satish Kavatagimath, Chanabasayya Vastrad, Shivakumar Kotrashetti

**Affiliations:** 1 Department of General Medicine, Basaveshwara Medical College, Chitradurga 577501, Karnataka, India; 2 Department of Pharmaceutical Chemistry, K.L.E. College of Pharmacy, Gadag 582101, Karnataka, India; 3 Department of Pharmacognosy, K.L.E. College of Pharmacy, Belagavi 590010, Karnataka, India; 4 Biostatistics and Bioinformatics, Chanabasava Nilaya, Bharthinagar, Dharwad 580001, Karnataka, India

**Keywords:** biomarker, GEO, subjects with diabetes and obesity, differentially expressed genes, pathways

## Abstract

*Background and Objectives:* A subject with diabetes and obesity is a class of the metabolic disorder. The current investigation aimed to elucidate the potential biomarker and prognostic targets in subjects with diabetes and obesity. *Materials and Methods:* The next-generation sequencing (NGS) data of GSE132831 was downloaded from Gene Expression Omnibus (GEO) database. Functional enrichment analysis of DEGs was conducted with ToppGene. The protein–protein interactions network, module analysis, target gene–miRNA regulatory network and target gene–TF regulatory network were constructed and analyzed. Furthermore, hub genes were validated by receiver operating characteristic (ROC) analysis. A total of 872 DEGs, including 439 up-regulated genes and 433 down-regulated genes were observed. *Results:* Second, functional enrichment analysis showed that these DEGs are mainly involved in the axon guidance, neutrophil degranulation, plasma membrane bounded cell projection organization and cell activation. The top ten hub genes (*MYH9*, *FLNA*, *DCTN1*, *CLTC*, *ERBB2*, *TCF4*, *VIM*, *LRRK2*, *IFI16* and *CAV1*) could be utilized as potential diagnostic indicators for subjects with diabetes and obesity. The hub genes were validated in subjects with diabetes and obesity. *Conclusion:* This investigation found effective and reliable molecular biomarkers for diagnosis and prognosis by integrated bioinformatics analysis, suggesting new and key therapeutic targets for subjects with diabetes and obesity.

## 1. Introduction

Diabetes mellitus and obesity are major metabolic or endocrine disorders and are dramatically increasing throughout the globe [1]. The prevalence of obesity and type 2 diabetes mellitus is considerably higher [2]. Diabetes mellitus and obesity are linked with progression of cardiovascular diseases [3], hypertension [4], and neurological and neuropsychiatric disorders [5] and asthma [6]. Till today, there is no cure for diabetes mellitus and obesity, and treatment and mediation tailored to clinical features are endorsed. Genetic and environmental factors are two initial contributors to these disorders [7]. Exploration of the molecular mechanisms of diabetes mellitus and obesity will develop the considerate of its pathogenesis and has key implications for designing new therapy.

Molecular mechanisms of subject with diabetes and obesity have been increasingly studied. Previous investigations showed that genes and signaling pathways are associated with diabetes mellitus and obesity. Key genes such as *ENPP1* [8] and *FTO* [9] were responsible for development of diabetes mellitus and obesity. Recent investigations showed that PI3K/AKT pathway [10] and TLR pathway [11] as a potential target for diabetes mellitus and obesity. However, certain key genes and pathways associated with diabetes mellitus and obesity have not been completely investigated. Further studies are necessary to elucidate these essential genes and pathways to provide novel therapeutic targets for the treatment of diabetes and obesity.

In recent years, the analysis of biological information, known as bioinformatics, has attracted a great deal of attention and sustained breakthroughs in the search for biomarkers for various diseases [12,13,14]. With the gradual advancement of next-generation sequencing (NGS) technology, bioinformatics has become increasingly essential in molecular pathogenesis, performing a major role in elucidating diseases mechanisms and finding novel targets for diseases treatment and patient prognosis [15]. With the wide function of NGS, a huge amount of data hasbeen generated, and most of the data have been deposited and stored in public databases. NGS data analyses have been carried out on diabetes and obesity in recent years [16], and hundreds of differentially expressed genes (DEGs) have been obtained. Bioinformatics methods combining with NGS techniques will be innovative.

**Figure 1:**
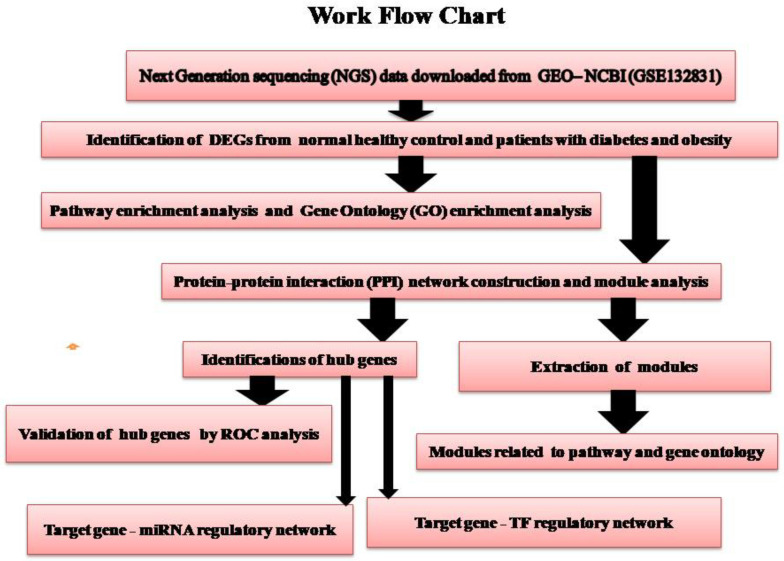
Overview of data analysis methodology.

Therefore, in this investigation, we downloaded the next-generation sequencing (NGS)data GSE132831, provided by Osinski et al. [17], from Gene Expression Omnibus (GEO, http://www.ncbi.nlm.nih.gov/geo/, accessed on 11 June 2020) [18] database to identify the differentially expressed genes (DEGs) between diabetes mellitus and obesity samples and normal control samples. With the identified DEGs, we performed Gene Ontology (GO) and pathway enrichment analyses to investigate the functions and pathways enriched by the DEGs. Additionally, we constructed a protein–protein interaction (PPI) network and modules screened out some important gene nodes to perform clustering analysis. Furthermore, we constructed a target gene–miRNA regulatory network and target gene–TF regulatory network based on these key genes to investigate the potential relationships between genes and subject with diabetes and obesity. Finally, hub genes were validated by using receiver operating characteristic (ROC) curve analysis. The research design of this study was shown in Figure 1. These results might provide novel ideas for future investigation and treatment of diabetes mellitus and obesity by exploring prognostic markers and therapeutic targets in diabetes mellitus and obesity.

## 2. Materials and Methods

### 2.1. RNA Sequencing Data

The NGS data GSE132831 was downloaded from the GEO database, which was based on the platform of GPL1857 Illumina NextSeq 500 (Homo sapiens). This dataset, including samples of 104 diabetic obese and samples of 120 normal control, was deposited by Osinski et al. [17].

### 2.2. Identification of DEGs

The limma R/Bioconductor software package was used to perform the identification of DEGs between samples of diabetic obese and normal control in R software [19]. The cutoff criteria were |logFC| > 1.112 for up-regulated genes, |logFC| <−0.64 for down-regulated genes, and a *p*-value < 0.05. The significance of *p* value measures how likely it is that any observed difference between two groups (diabetes mellitus and obesity samples and normal control samples). The significance of log FC looks only at genes which vary wildly amongst other genes.

### 2.3. GO and Pathway Enrichment Analyses of DEGs

The ToppGene (ToppFun) (https://toppgene.cchmc.org/enrichment.jsp, accessed on 11 June 2020) [20] bioinformatics resources was utilized to distinguish and enrich the biological attributes, such as biological processes (BP), cellular components (CC), molecular functions (MF) and pathways, of identified DEGs (Up- and down-regulated genes separately). Moreover, GO (http://geneontology.org/, accessed on 11 June 2020) [21] and REACTOME (https://reactome.org/, accessed on 11 June 2020) [22] pathway enrichment analyses were used to identify the significant GO terms and pathways. *p* < 0.05 was set as the cutoff criterion for significant enrichment.

### 2.4. Protein–Protein Interaction (PPI) Network and Module Analysis

The IID interactome (http://iid.ophid.utoronto.ca/, accessed on 11 June 2020) [23] is an online database containing known and predicted PPI networks. In this investigation, a PPI network of identified DEGs in dataset was identified using the IID interactome database (combined score >0.4) and subsequently visualized using Cytoscape (http://www.cytoscape.org/, accessed on 11 June 2020) software (version 3.8.2) [24]. The regulatory relationship between genes were analyzed through topological property of computing network including the node degree [25], betweenness centrality [26], stress centrality [27] and closeness centrality [28] by using the Network Analyzer app within Cytoscape. The PEWCC1 (http://apps.cytoscape.org/apps/PEWCC1, accessed on 11 June 2020) [29] program within Cytoscape was used to detect modules of the PPI network. The GO and pathway enrichment analysis of the identified modules was then performed using the ToppGene database.

### 2.5. Target Gene–miRNARegulatory Network

miRNet database (https://www.mirnet.ca/, accessed on 11 June 2020) [30] is a bioinformatics platform for predicting target gene–miRNA pairs. In the present study, the target genes were predicted using 14 miRNA databases: TarBase, miRTarBase, miRecords, miRanda (S mansoni only), miR2Disease, HMDD, PhenomiR, SM2miR, PharmacomiR, EpimiR, starBase, TransmiR, ADmiRE, and TAM 2.0. In this study, miRNAs were considered the targeted miRNAs of hub genes based on these miRNA databases. The target gene–miRNA regulatory network was depicted and visualized using Cytoscape software.

### 2.6. Target Gene–TF Regulatory Network

NetworkAnalyst database (https://www.networkanalyst.ca/, accessed on 11 June 2020) [31] is a bioinformatics platform for predicting target gene–TF pairs. In the present study, the target genes were predicted using ChEA TF database. In this study, TFs were considered the targeted TFs of hub genes based on this TF database. The target gene–TF regulatory network was depicted and visualized using Cytoscape software.

### 2.7. Receiver Operating Characteristic (ROC) Analysis

A ROC analysis is a technique for visualizing, construct and determining classifiers based on their achievement. A diagnostic test was firstly performed in order to measure the diagnostic value of candidate biomarkers in subject with diabetes and obesity. Sensitivity and specificity of each biomarker in this diagnostic test were determined. ROC curves were retrieved by plotting the sensitivity, against the specificity using the pROC in R software [32]. Area under the ROC curve (AUC) was determined to predict the efficiency of this diagnostic test. A test with AUC bigger than 0.9 is assigned great efficiency, 0.7–0.9, modest efficiency and 0.5–0.7, small efficiency.

## 3. Results

### 3.1. Identification of DEGs

**Figure 2:**
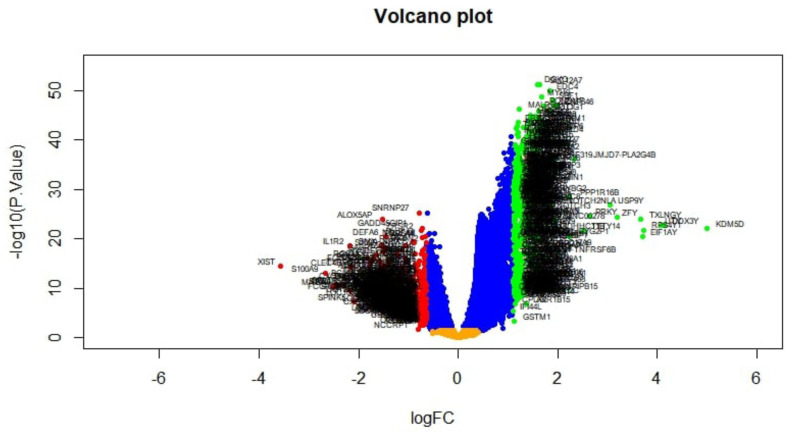
Volcano plot of differentially expressed genes. Genes with a significant change of more than two-fold were selected. Green dot represented up-regulated significant genes and red dot represented down-regulated significant genes.

**Figure 3:**
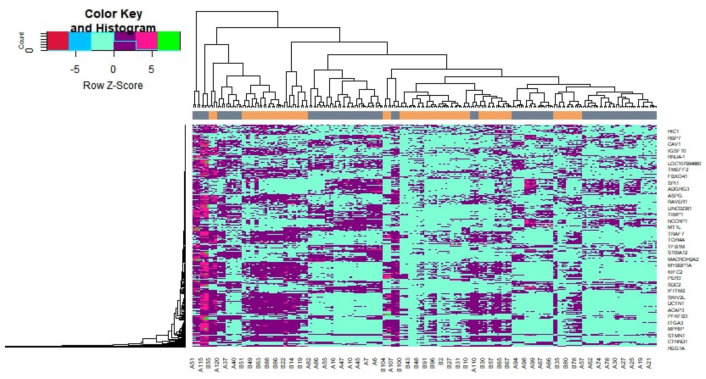
Heat map of differentially expressed genes. Legend on the top left indicate log fold change ingenes. (A1–A120 = non-diabetic obese samples; B1–B104 = diabetic obese samples.)

The DEGs were screened by “limma” package (*p*-value < 0.05, and |logFC| > 1.112 for up-regulated genes and |logFC| <−0.64 for down-regulated genes). The GSE132831 dataset contained 872 DEGs, including 439 up-regulated genes and 433 down-regulated genes. DEGs are listed in Table S1. The volcano plot is presented in Figure 2. The heat map DEGs is shown in Figure 3.

### 3.2. GO and Pathway Enrichment Analyses of DEGs

To gain in-depth and comprehensive biological characteristics of these DEGs, GO functional annotation and REACTOME pathway enrichment analysis were performed through online analytical tool ToppGene. The BP was mainly enriched in plasma membrane bounded cell projection organization, neurogenesis, cell activation and secretion (Table S2). The CC was mainly enriched in neuron projection, golgi apparatus, secretory granule and secretory vesicle (Table S2). The MF was significantly enriched in drug binding, ribonucleotide binding, signaling receptor binding and molecular transducer activity (Table S2). Result of REACTOME enrichment analysis showed that top pathways were axon guidance, extracellular matrix organization, neutrophil degranulation and innate immune system (Table S3).

### 3.3. Protein–Protein Interaction (PPI) Network and Module Analysis

**Figure 4:**
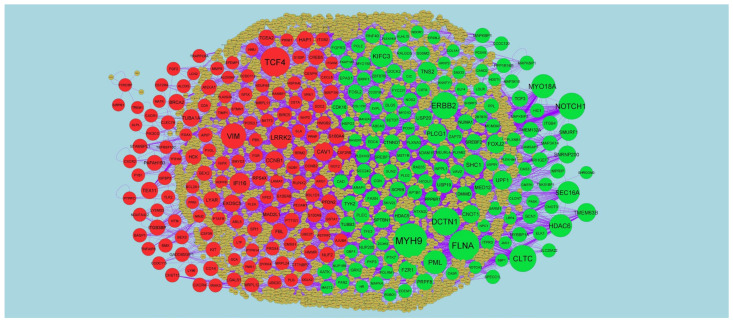
PPI network of DEGs. The PPI network of DEGs was constructed using Cytoscap. Up-regulated genes are marked in green; down-regulated genes are marked in red.

**Figure 5:**
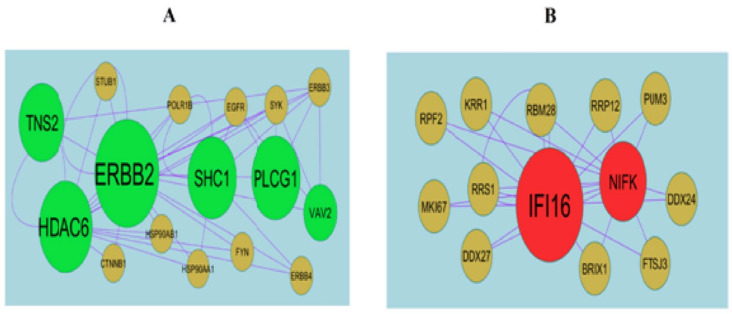
Modules of isolated form PPI of DEGs. (**A**) The most significant module was obtained from PPI network with 16 nodes and 39 edges for up-regulated genes. (**B**) The most significant module was obtained from PPI network with 13 nodes and 24 edges for down-regulated genes. Up-regulated genes are marked in green; down-regulated genes are marked in red.

To find the hub genes in the DEGs, Network Analyzer, a plug-in Cytoscape was performed. All the genes and edges were determined. IID interactome mapped 872 DEGs into a PPI network containing 3894 nodes and 7142 edges (Figure 4). Hub genes with the high node degree, betweenness centrality, stress centrality and closeness centrality are listed in Table S4. MYH9 (Degree 231; Betweenness 0.083106; Stress 11909200; Closeness 0.348923), FLNA (Degree 196; Betweenness 0.07999; Stress 10927852; Closeness 0.35285), DCTN1 (Degree 168; Betweenness 0.080808; Stress 7748054; Closeness 0.330668), CLTC (Degree 158; Betweenness 0.071579; Stress 7687192; Closeness 0.351161), ERBB2 (Degree 158; Betweenness 0.069347; Stress 7857692; Closeness 0.327216), TCF4 (Degree 186; Betweenness 0.080443; Stress 7982798; Closeness 0.320102), VIM (Degree 146; Betweenness 0.062238; Stress 9673084; Closeness 0.327078), LRRK2 (Degree 114; Betweenness 0.046449; Stress 5881270; Closeness 0.333305), IFI16 (Degree 91; Betweenness 0.035934; Stress 2890936; Closeness 0.294671) and CAV1 (Degree 74; Betweenness 0.034501; Stress 3642486; Closeness 0.323593). Then, PEWCC1 was used to find clusters in the network. Module1 contained 16 nodes and 39 edges (Figure 5A). Module1 was associated with including axon guidance, signaling by NGF, plasma membrane bounded cell projection organization and neurogenesis. Module2contained 13 nodes and 24 edges (Figure 5B). Module 2 was associated with an innate immune system.

### 3.4. Target Gene–miRNA Regulatory Network

**Figure 6:**
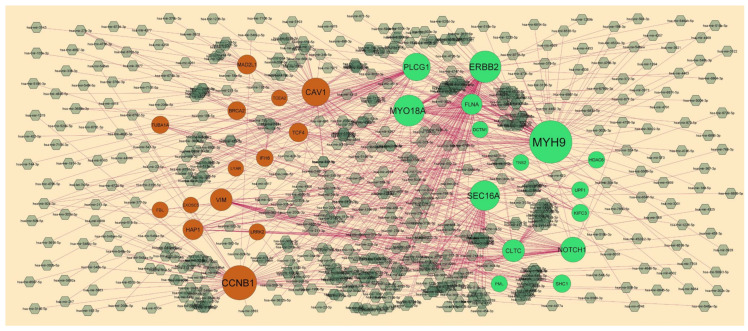
Target gene–miRNA regulatory network between target genes. The black-color diamond nodes represent the key miRNAs; up-regulated genes are marked in green; down-regulated genes are marked in orange.

The target gene–miRNA regulatory network included 2520 nodes (miRNAs: 2224; gene: 296) and 15485 edges (Figure 6). The nodes with degrees were listed in Table S5. We discovered that MYH9 was targeted by 116 miRNAs (ex; hsa-mir-4329); ERBB2 was targeted by 73miRNAs (ex; hsa-mir-4315); MYO18A was targeted by 71miRNAs (ex; hsa-mir-1299); SEC16A was targeted by 66 miRNAs (ex; hsa-mir-4779); PLCG1 was targeted by 56 miRNAs (ex; hsa-mir-3685); CCNB1 was targeted by 84miRNAs (ex; hsa-mir-6134); CAV1 was targeted by 58miRNAs (ex; hsa-mir-4459); VIM was targeted by 30miRNAs (ex; hhsa-mir-6124); HAP1 was targeted by 22miRNAs (ex; hsa-mir-9500); MAD2L1 was targeted by 17miRNAs (ex; hsa-mir-1297).

### 3.5. Target Gene–TF Regulatory Network

**Figure 7:**
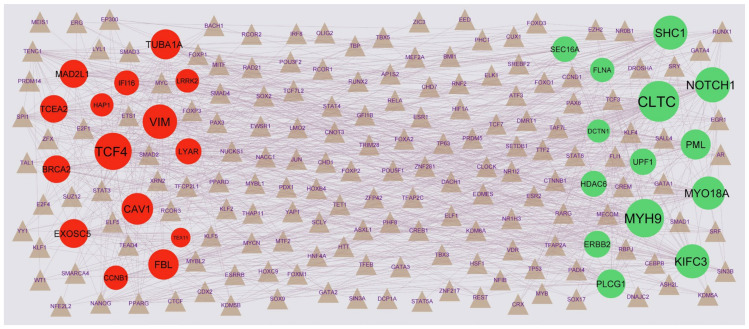
Target gene–TF regulatory network between target genes. The gray-color triangle nodes represent the key TFs; up-regulated genes are marked in green; down-regulated genes are marked in red.

The target gene–TF regulatory networkincluded 487 nodes (TFs: 195; gene: 292) and 7094 edges (Figure 7). The nodes with degrees were listed in Table S5. We discovered that that CLTC was targeted by 59 TFs (ex; SMARCA4); MYH9 was targeted by 53 TFs (ex; TCF7); NOTCH1 was targeted by 45 TFs (ex; MYB); SHC1 was targeted by 42 TFs (ex; E2F4); KIFC3 was targeted by 42 TFs (ex; CUX1); TCF4 was targeted by 50 TFs (ex; NANOG); VIM was targeted by 43 TFs (ex; GFI1B); CAV1 was targeted by 36 TFs (ex; GATA4); FBL was targeted by 33 TFs (ex; HIF1A); TUBA1A was targeted by 32 TFs (ex; CLOCK).

### 3.6. Receiver Operating Characteristic (ROC) Analysis

**Figure 8:**
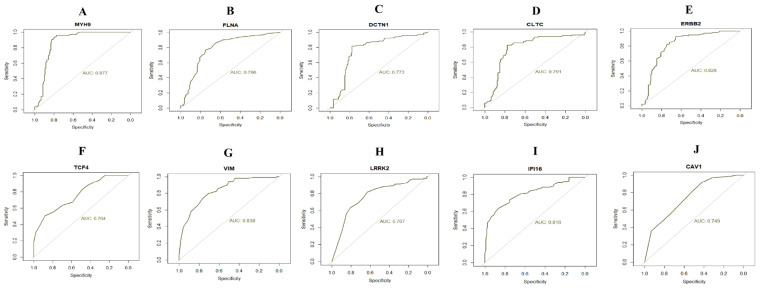
ROC curve analyses of hub genes. (**A**) MYH9 (**B**) FLNA (**C**) DCTN1 (**D**) CLTC (**E**) ERBB2 (**F**) TCF4 (**G**) VIM (**H**) LRRK2 (**I**) IFI16 (**J**) CAV1.

To identify new potential biomarkers for diabetes and obesity, ROC curves of data derived from healthy controls and patients with diabetes and obesity was analyzed using the R package. The AUC calculated to assess the discriminatory ability of hub genes (Figure 8). Validated by ROC curves, we found that hub genes had high sensitivity and specificity, including *MYH9*, *FLNA*, *DCTN1*, *CLTC*, *ERBB2*, *TCF4*, *VIM*, *LRRK2*, *IFI16* and *CAV1*, and AUC values more than 0.7. This analysis demonstrated that the hub genes had a diagnostic role.

## 4. Discussion

A NGS investigation is an ideal way to comprehensively investigate diabetes mellitus and obesity. In this investigation, we collected NGS dataset from the GEO database, and a total of 872 DEGs, including 439 up-regulated genes and 433 down-regulated genes, were found. Altered expression of *XIST* (X inactive specific transcript) [33] and *SELL* (selectin L) [34] are associated with prognosis ofdiabetes. *S100A9* and *S100A8* are associated with the prognosis of diabetes mellitus and obesity [35]. *IL1R2* [36] and *SPINK5* [37] plays an important role in the diabetes mellitus and obesity.

GO term and REACTOME enrichment analyzes were accomplished to examine interactions between the DEGs. The altered expression of genes including *ERBB2* [38], *DACT1* [39], *ARAP1* [40], *MYH9* [41], *INPPL1* [42], *SARM1* [43], *NOTCH1* [44], *ROBO1* [45], *MAPK8IP1* [46], *ANK1* [47], *SARM1* [43], *SREBF2* [48], *SIK1* [49], *PASK* (PAS domain-containing serine/threonine kinase) [50], *NOS2* [51], *OAS3* [52], *KL* (klotho) [53], *PECAM1* [54], *S100A12* [55], *S100P* [56], *BATF3* [57], *PLEK* (pleckstrin) [58], *ALOX5* [59], *ARG1* [60], *CXCL8* [61], *CXCR1* [62], *PTAFR* (platelet-activating factor receptor) [63], *PYGL* (glycogen phosphorylase L) [64], *TCF4* [65], *CAMP* (cathelicidin antimicrobial peptide) [66], *RUNX2* [67], *PLA2G2A* [68], *GCG* (glucagon) [69], *RARRES2* [70] and *HAP1* [71] in diabetes mellitus was reported to be an independent prognostic factors. *ACHE* (acetylcholinesterase) [72], *FGFR3* [73], *VLDLR* (very-low-density lipoprotein receptor) [74], *SHC1* [75], *HDAC6* [76], *CHRNA2* [77], *CASR* (calcium-sensing receptor) [78], *ELK1* [79], *TYK2* [80], *CIITA* (class II major histocompatibility complex transactivator) [81], *ZAP70* [82], *GPT* (glutamic-pyruvic transaminase) [83], *CHI3L1* [84], *AIF1* [85], *MMP9* [86], *ITGB2* [87], *CFD* (complement factor D) [88], *C3AR1* [89], *LGALS1* [90], *CD14* [91], *TIMP1* [92], *TLR2* [93], *LTF* (lactotransferrin) [94], *BRCA2* [95] and *IGFBP3* [96] are a potential prognostic markers in obesity. Sun et al. [97] reported that *TRPM2* was significantly regulated in diabetes and obesity. Findings were implied by Richter et al. [98], Suchkova et al. [99], Qureshi et al. [100], Wang et al. [101], Wang et al. [102], Aoki-Suzuki et al. [103], Ohno et al. [104], Richter et al. [98], Rahman and Copeland [105], Congiu et al. [106], Ji et al. [107], Wollmer et al. [108], Yamazaki et al. [109], Bardien et al. [110], Comella Bolla et al. [111], Horvath et al. [112], Watanabe et al. [113], Kushima et al. [114], Grünblatt et al. [115], and Sato and Kawata [116] when they found that *TAOK2*, *ACAP3*, *PLXNA3*, *PLXNA4*, *DCTN1*, *NTNG2*, *LRP4*, *AGRN* (agrin), *TAOK2*, *POLG* (DNA polymerase gamma, catalytic subunit), *KCNK2*, *OPRK1*, *ABCA2*, *ABCA7*, *LRRK2*, *CD200*, *PAK3*, *PADI2*, *EPHB1*, *CHAT* (choline O-acetyltransferase) and *SLC18A1* plays a substantial role in the patients with neurological and neuropsychiatric disorders. Studies showed that biomarkers include *PLD2* [117], *FLNA* (filamin A) [118], *SMURF1* [119], *LINGO1* [120], *CACNA1H* [121], *NLRP6* [122], *NLRC3* [123], *CXCR2* [124] and *C5AR1* [125] plays an important role in progression of hypertension. Sauzeau et al. [126], Xu et al. [127], Hirota et al. [128], Alharatani et al. [129], Beitelshees et al. [130], Zhu et al. [131], Gil-Cayuela et al. [132], Liu et al. [133], Xie et al. [134], Kroupis et al. [135], López-Mejías et al. [136], Gremmel et al. [137] Yamada and Guo [138], Petri et al. [139], DeFilippis et al. [140], Rocca et al. [141] and Tur et al. [142] found that genes include *VAV2*, *RASAL1*, *LIF* (LIF interleukin 6 family cytokine), *CTNND1*, *CACNA1C*, *MAP3K10*, *NRBP2*, *TRPM4*, *LILRB2*, *FCGR2A*, *PIK3CG*, *SELPLG* (selectin P ligand), *PRDX4*, *FPR2*, *PLG* (plasminogen), *SELENOM* (selenoprotein M) and *NCAM1* were a diagnostic markers of cardiovascular diseasesand could be used as therapeutic targets. Accumulating evidence shows that *ITGB4* [143], *SEMA3D* [144], *FCAR* (Fc fragment of IgA receptor) [145], *KIT* (KIT proto-oncogene, receptor tyrosine kinase) [146], *PGLYRP1* [147], *IL17RB* [148], *BIRC5* [149] and *PTGS1* [150] are associated with prognosis in asthma. Studies showed that *GRK2* [151], *ADCY3* [152], *FASN* (fatty acid synthase) [153], *DGKD* (diacylglycerol kinase delta) [154], *DGKQ* (diacylglycerol kinase theta) [154], *IP6K1* [155], *ANXA1* [156], *SUCNR1* [157], *PRNP* (prion protein) [158], *CXCR4* [159], *CAV1* [160], *LCN2* [161], *AQP9* [162], *NMU* (neuromedin U) [163], *NPY1R* [164], *FFAR2* [165], *OSM* (oncostatin M) [166] and *TREM1* [167] might be a potential markers for diabetes mellitus and obesity. Researchers have shown that *UNC13B* [168], *PFKFB3* [169], *FCN1* [170] and *SLC11A1* [171] were diagnostic markers for type 1 diabetes. DEGs involved in GO terms and pathways were more likely related to diabetes mellitus and obesity, and DEGs also involved in neurological and neuropsychiatric disorders, hypertension, cardiovascular diseases and asthma.

As known, dynamic networks analysis and disease gene association were criteria for progression of various diseases [172,173]. Protein–protein interaction (PPI) network and its module can be regarded as key to the understanding of progression of diabetes mellitus and obesity, and might also lead to novel therapeutic way. *MYH9* [41,174,175,176], *ERBB2* [38,177,178,179,180], *TCF4* [65,181], *VIM* (vimentin) [182,183], *LRRK2* [184,185] and *CAV1* [161,186,187,188,189,190,191,192] have been implicated as a principal mediator of diabetes mellitus. *VIM* (vimentin) binds to insulin-responsive aminopeptidas, a major cargo protein of glucose transporter type 4, and decreases the glucose tolerance [182]. *IFI16* [193], *ERBB2* [194], *VIM* (vimentin) [182,195] and *CAV1* [160,196,197,198,199] are crucial factors for advancement of obesity. IFI16 showed adipogenesis, an enhanced inflammatory state and damaged insulin-stimulated glucose uptake in adipose tissue [193]. Motor protein MYH9 bindsto actin and producesmechanical force through magnesium-dependent hydrolysis of ATP, and it generatesthe contraction of striated and smooth muscles [200]. ErbB2 is a receptor tyrosine kinase family whose activity in cells depends on dimerization with another ligand-binding ErbB receptor, and associated with progression of various diseases [201]. TCF4 is a member of the basic helix–loop–helix (bHLH) family of transcription factors that have a key role in a various diseases [202]. VIM (vimentin) is an intermediate filament (IF) protein and plays an important role in epithelial–mesenchymal transition (EMT), a process that occurs during the development of various diseases [203]. *LRRK2* is an enigmatic protein and has been one of the central molecules in a number of human diseases [204]. CAV1 is a cell surface protein shownto play a key role in insulin resistance [205]. IFI16 is an innate immune sensor for intracellular DNA and is associated with DNA damage in various diseases [206]. We identified novel targets including *CLTC* (clathrin heavy chain), *TNS2*, *PLCG1* and *NIFK* (nucleolar protein interacting with the FHA domain of MKI67) for specific therapy of diabetes mellitus and obesity. Further investigation is needed to validate these results and investigate the roles of these biomarkers in diabetes mellitus and obesity.

In the present investigation, NGS data analysis revealed that the mechanism of occurrence of diabetes mellitus and obesity might be related to the expression of miRNA and TF. To validate the accuracy of the target genes, miRNAs and TFs identified by target gene–miRNA regulatory network and target gene–TF regulatory network analysis. Yan et al. [207], Wang et al. [208], Yan et al. [209] and Guo et al. [210] showed that expression and prognosis of *hsa-mir-4329*, *hsa-mir-3685*, *hsa-mir-6124*, *hsa-mir-1297* and *SMARCA4* are associated with the risk of cardiovascular diseases. Several studies have shown that biomarkers including *hsa-mir-1299* [211], *hsa-mir-4779* [212] and *hsa-mir-4459* [213] might be predictive biomarkers for the efficacy of diabetes mellitus treatment. *TCF7* was revealed and regarded as diagnostic biomarker in type 1 diabetes mellitus [214]. Transcription factor *MYB* was involved in asthma [215]. *MYB* might be associated with diabetes and obesity. *E2F4* [216] and *CLOCK* [217] are associated with prognosis in patients with diabetes mellitus and obesity. *CUX1* [218], *NANOG* [219], *GATA4* [220] and *HIF1A* [221] plays a vital role in the patients with obesity. Novel targets include *MYO18A*, *SEC16A*, *CCNB1*, *MAD2L1*, *hsa-mir-4315*, *hsa-mir-6134*, *hsa-mir-9500*, *KIFC3*, *FBL* (fibrillarin), *TUBA1A* and *GFI1B* might have crucial biologic functions in the pathogenesis of patients with diabetes mellitus and obesity. This result indicated that our identified biomarkers are involved in the pathological progression of diabetes and obesity, its associated complications beingneurological and neuropsychiatric disorders, hypertension, cardiovascular diseases and asthma, thus warranting further exploration.

However, there are some limitations in this investigation. For instance, the NGS data were obtained from the GEO database and were not given by the authors. Therefore, further research should be conducted to verify whether these target genes can be used in the clinical treatment of diabetes mellitus and obesity.

## 5. Conclusions

Using a bioinformatics analysis of NGS dataset GSE132831, we identified the genes of diabetes and obesity. We found that DEGs in patients were enriched for pathways mainly involved in the axon guidance, neutrophil degranulation, plasma membrane-bounded cell projection organization, and cell activation. Focusing on the key genes and corresponding pathways involved in diabetes and obesity could provide new insights for diabetes mellitus and obesity treatment. Hub genes including MYH9, FLNA, DCTN1, CLTC, ERBB2, TCF4, VIM, LRRK2, IFI16 and CAV1 were identified as potential novel biomarkers for diabetes and obesity. The validation of hub genes was demonstrated by ROC analysis. Further investigation isurgently demanded to validate the hub genes, and further molecular mechanisms would be uncovered. All the output will lay the foundation for finding a possible therapeutic strategy to treat diabetes mellitus and obesity.

## Data Availability

The datasets supporting the conclusions of this article are available in the GEO (Gene Expression Omnibus) (https://www.ncbi.nlm.nih.gov/geo/, accessed on 11 June 2020) repository. [(GSE132831) (https://www.ncbi.nlm.nih.gov/geo/query/acc.cgi?acc=GSE132831, accessed on 11 June 2020)].

## Supplementary Materials

The following supporting information can be downloaded at: https://www.mdpi.com/article/10.3390/medicina59020309/s1, Table S1: The statistical metrics for key differentially expressed genes (DEGs).; Table S2: The enriched GO terms of the up and down regulated differentially expressed genes; Table S3: The enriched pathway terms of the up and down regulated differentially expressed genes; Table S4: Topology table for up and down regulated genes; Table S5: miRNA—target gene and TF—target gene interaction.
