# Supplementary material for: Bioinformatics and Next-Generation Data Analysis for Identification of Genes and Molecular Pathways Involved in Subjects with Diabetes and Obesity
Source: Medicina (Kaunas). 2023 Feb 7;59(2):309. doi: 10.3390/medicina59020309 (PMC9967176; doi:10.3390/medicina59020309)
Supplement: Supplementary file 1 [file medicina-59-00309-s001.zip › medicina-2143361-supplementary.pdf]

Table S1. The statistical metrics for key differentially expressed genes (DEGs).

Gene Symbol	logFC	pValue	adj.P.Val	tvalue	Regulation	Gene Name
KDM5D	5.006563	8.7E-23	3.29E-22	10.97252	Up	lysine demethylase 5D
DDX3Y	4.136584	2.21E-23	9.1E-23	11.16119	Up	DEAD-box helicase 3 Y-linked
UTY	4.070223	2.16E-23	8.91E-23	11.16417	Up	ubiquitously transcribed tetratricopeptide repeat containing, Y-linked
RPS4Y1	3.719159	1.61E-22	5.97E-22	10.88712	Up	ribosomal protein S4 Y-linked 1
EIF1AY	3.710641	3.69E-21	1.24E-20	10.45171	Up	eukaryotic translation initiation factor 1A Y-linked
TXLNGY	3.657216	1.09E-24	5E-23	11.57187	Up	taxilin gamma pseudogene, Y-linked
ZFY	3.196376	3.56E-25	3.97E-23	11.72394	Up	zinc finger protein Y-linked
USP9Y	3.054299	1.7E-27	2.74E-25	12.44369	Up	ubiquitin specific peptidase 9 Y-linked
PRKY	2.646358	2.69E-25	3.1E-23	11.76204	Up	protein kinase Y-linked (pseudogene)
TTTY14	2.513856	2.38E-22	8.66E-22	10.83353	Up	testis-specific transcript, Y-linked 14
JMJD7-PLA2G4B	2.341619	7.14E-37	4.24E-34	15.29814	Up	JMJD7-PLA2G4B readthrough
GYG2P1	2.233505	2.84E-21	9.57E-21	10.48845	Up	glycogenin 2 pseudogene 1
PPP1R16B	2.224558	2.69E-29	5.59E-27	12.99622	Up	protein phosphatase 1 regulatory subunit 16B
NPIPB15	1.987655	3.06E-10	5.77E-10	6.585861	Up	nuclear pore complex interacting protein family member B15
ZNF646	1.979401	1.2E-47	8.69E-44	18.59107	Up	zinc finger protein 646
ZNF319	1.951946	6.77E-37	4.11E-34	15.30502	Up	zinc finger protein 319
LINC00278	1.936282	2.21E-24	5E-23	11.47602	Up	long intergenic non-protein coding RNA 278
NSMF	1.918356	7.56E-48	7.33E-44	18.65279	Up	NMDA receptor synaptonuclearsignaling and neuronal migration factor
SBF1	1.903964	8.22E-49	9.56E-45	18.9521	Up	SET binding factor 1
NOTCH2NLA	1.889919	1.37E-27	2.27E-25	12.47183	Up	notch 2 N-terminal like A
PLCG1	1.859398	1.47E-46	8.01E-43	18.25362	Up	phospholipase C gamma 1
EDC4	1.850651	9.13E-51	1.77E-46	19.56249	Up	enhancer of mRNA decapping 4
EVPL	1.838017	3.97E-42	6.42E-39	16.89446	Up	envoplakin
SLC17A9	1.828051	5.2E-19	1.53E-18	9.750293	Up	solute carrier family 17 member 9
ZDHHC11B	1.827313	1.75E-22	6.43E-22	10.87596	Up	zinc finger DHHC-type containing 11B
HPCAL4	1.817644	5.65E-19	1.66E-18	9.738261	Up	hippocalcin like 4
AATK	1.811724	3.21E-44	9.33E-41	17.53451	Up	apoptosis associated tyrosine kinase
COL1A1	1.803059	6.32E-13	1.35E-12	7.628451	Up	collagen type I alpha 1 chain
NOTCH3	1.774964	1.94E-26	2.71E-24	12.11706	Up	notch receptor 3
GPRIN1	1.745359	3.1E-32	9.21E-30	13.89188	Up	G protein regulated inducer of neurite outgrowth 1
SIK1	1.739946	8.18E-17	2.08E-16	9.011793	Up	salt inducible kinase 1

CRYBG2	1.726069	3.85E-30	8.88E-28	13.2545	Up	crystallin beta-gamma domain containing 2
POLE	1.718958	1E-47	8.34E-44	18.61453	Up	DNA polymerase epsilon, catalytic subunit
WDR81	1.703126	9.68E-44	2.56E-40	17.38756	Up	WD repeat domain 81
MYH9	1.676655	1.57E-49	2.29E-45	19.17585	Up	myosin heavy chain 9
DAW1	1.668583	4.26E-10	7.99E-10	6.527126	Up	dynein assembly factor with WD repeats 1
SLC9A3	1.661086	3.3E-25	3.73E-23	11.73409	Up	solute carrier family 9 member A3
PITPNM1	1.655779	2.31E-44	7.47E-41	17.57815	Up	phosphatidylinositol transfer protein membrane associated 1
ROBO1	1.655184	2.55E-13	5.51E-13	7.774736	Up	roundabout guidance receptor 1
INTS1	1.647598	1.5E-43	3.8E-40	17.32893	Up	integrator complex subunit 1
NYNRIN	1.639731	1.76E-25	2.15E-23	11.81962	Up	NYN domain and retroviral integrase containing
SLC12A7	1.635974	6.25E-52	1.82E-47	19.92856	Up	solute carrier family 12 member 7
ZNF598	1.632571	2.29E-33	8.02E-31	14.23559	Up	zinc finger protein 598
PPP6R1	1.629126	5.04E-45	1.83E-41	17.78112	Up	protein phosphatase 6 regulatory subunit 1
COL18A1	1.621947	7.18E-16	1.75E-15	8.686496	Up	collagen type XVIII alpha 1 chain
GRAMD4	1.62178	5.99E-42	9.41E-39	16.84019	Up	GRAM domain containing 4
MAPK8IP1	1.610516	1.34E-20	4.38E-20	10.27046	Up	mitogen-activated protein kinase 8 interacting protein 1
MAN2A2	1.609577	1.68E-38	1.24E-35	15.79199	Up	mannosidase alpha class 2A member 2
CNOT1	1.607611	1.51E-46	8.01E-43	18.25007	Up	CCR4-NOT transcription complex subunit 1
CACNA1H	1.606751	6.35E-21	2.11E-20	10.37568	Up	calcium voltage-gated channel subunit alpha1 H
TAF1C	1.603583	1.21E-36	6.68E-34	15.22922	Up	TATA-box binding protein associated factor, RNA polymerase I subunit C
DMBT1	1.60288	3.46E-15	8.17E-15	8.447147	Up	deleted in malignant brain tumors 1
SEZ6	1.598893	7.45E-19	2.17E-18	9.698622	Up	seizure related 6 homolog
DGKQ	1.593962	4.49E-52	1.82E-47	19.97375	Up	diacylglycerol kinase theta
ARVCF	1.592098	9.55E-18	2.54E-17	9.328467	Up	ARVCF delta catenin family member
MST1R	1.591148	6.15E-43	1.19E-39	17.1419	Up	macrophage stimulating 1 receptor
OPLAH	1.590504	2.18E-43	5.08E-40	17.27938	Up	5-oxoprolinase, ATP-hydrolysing
ZNF316	1.5893	6.1E-38	4.08E-35	15.62201	Up	zinc finger protein 316
HSPG2	1.58419	9.22E-35	3.94E-32	14.65846	Up	heparansulfate proteoglycan 2
ZDHHC8	1.572303	1.83E-28	3.46E-26	12.74083	Up	zinc finger DHHC-type containing 8
PPP1R26	1.572208	3.07E-36	1.58E-33	15.10616	Up	protein phosphatase 1 regulatory subunit 26
ITPR3	1.567244	4.26E-39	3.59E-36	15.97248	Up	inositol 1,4,5-trisphosphate receptor type 3
DCHS1	1.558789	2.54E-20	8.22E-20	10.1802	Up	dachsous cadherin-related 1
MYO18A	1.558693	6.7E-43	1.26E-39	17.1305	Up	myosin XVIIIA
PHRF1	1.557745	1.72E-45	7.68E-42	17.92503	Up	PHD and ring finger domains 1

NTNG2	1.555737	3.21E-18	8.82E-18	9.487351	Up	netrin G2
MYO7B	1.554441	2.13E-42	3.65E-39	16.97705	Up	myosin VIIB
OPRK1	1.552814	1.2E-12	2.51E-12	7.524967	Up	opioid receptor kappa 1
CHPF2	1.550982	1.03E-38	7.81E-36	15.85577	Up	chondroitin polymerizing factor 2
FLNA	1.548865	4.51E-29	9.14E-27	12.92759	Up	filamin A
SREBF2	1.54358	4.83E-39	3.9E-36	15.95617	Up	sterol regulatory element binding transcription factor 2
KIFC2	1.541309	1.4E-34	5.78E-32	14.60334	Up	kinesin family member C2
ZSWIM8	1.540738	4.22E-45	1.64E-41	17.80475	Up	zinc finger SWIM-type containing 8
SGSM2	1.531883	3.14E-39	2.77E-36	16.01293	Up	small G protein signaling modulator 2
AMIGO1	1.531298	1.92E-21	6.52E-21	10.54327	Up	adhesion molecule with Ig like domain 1
NLRC3	1.531139	1.33E-21	4.57E-21	10.59421	Up	NLR family CARD domain containing 3
TUBGCP6	1.528464	1.41E-42	2.57E-39	17.0314	Up	tubulin gamma complex associated protein 6
SP6	1.528296	1.6E-23	6.8E-23	11.2051	Up	Sp6 transcription factor
ZBTB7B	1.528224	9.08E-38	5.94E-35	15.56952	Up	zinc finger and BTB domain containing 7B
CAPN15	1.524929	4.69E-41	5.57E-38	16.56774	Up	calpain 15
MED12L	1.523987	7.69E-15	1.78E-14	8.324426	Up	mediator complex subunit 12L
INPPL1	1.523926	6.77E-40	6.91E-37	16.21522	Up	inositol polyphosphate phosphatase like 1
NUMA1	1.523239	3.72E-41	4.51E-38	16.59843	Up	nuclear mitotic apparatus protein 1
DAGLA	1.521846	3.22E-17	8.33E-17	9.15008	Up	diacylglycerol lipase alpha
PLCH2	1.515783	6.41E-26	8.43E-24	11.95576	Up	phospholipase C eta 2
RAP1GAP	1.51542	1.51E-18	4.27E-18	9.596633	Up	RAP1 GTPase activating protein
SCRIB	1.508891	1.57E-34	6.38E-32	14.5886	Up	scribble planar cell polarity protein
HLF	1.508651	1.32E-15	3.19E-15	8.594222	Up	HLF transcription factor, PAR bZIP family member
DNAH1	1.507391	2.26E-39	2.12E-36	16.05604	Up	dynein axonemal heavy chain 1
LINC02593	1.503404	4.78E-12	9.76E-12	7.297261	Up	long intergenic non-protein coding RNA 2593
GCN1	1.501349	1.12E-41	1.59E-38	16.75759	Up	GCN1 activator of EIF2AK4
LIME1	1.497861	8.22E-21	2.72E-20	10.33945	Up	Lck interacting transmembrane adaptor 1
LIF	1.497851	2.41E-27	3.81E-25	12.39667	Up	LIF interleukin 6 family cytokine
IL12RB2	1.492097	2.56E-15	6.1E-15	8.493085	Up	interleukin 12 receptor subunit beta 2
ARAP1	1.491472	1.93E-41	2.49E-38	16.68557	Up	ArfGAP with RhoGAP domain, ankyrin repeat and PH domain 1
HR	1.491421	5.28E-29	1.07E-26	12.90664	Up	HR lysine demethylase and nuclear receptor corepressor
SSH3	1.490041	7.4E-41	8.61E-38	16.50759	Up	slingshot protein phosphatase 3
MAN2C1	1.489744	2.99E-37	1.89E-34	15.41256	Up	mannosidase alpha class 2C member 1
BAIAP3	1.48829	9.98E-18	2.65E-17	9.322013	Up	BAI1 associated protein 3

DLG5	1.486894	6.34E-42	9.71E-39	16.83256	Up	discs large MAGUK scaffold protein 5
USP20	1.48295	1.38E-44	4.71E-41	17.64723	Up	ubiquitin specific peptidase 20
GABRR2	1.478466	6.54E-19	1.92E-18	9.717314	Up	gamma-aminobutyric acid type A receptor rho2 subunit
ACHE	1.478366	4.44E-39	3.66E-36	15.96712	Up	acetylcholinesterase (Cartwright blood group)
NCR3LG1	1.478315	2.93E-21	9.89E-21	10.48385	Up	natural killer cell cytotoxicity receptor 3 ligand 1
FAM83G	1.475383	1.52E-36	8.28E-34	15.19836	Up	family with sequence similarity 83 member G
MAPK8IP3	1.466652	1.06E-34	4.47E-32	14.64015	Up	mitogen-activated protein kinase 8 interacting protein 3
AVPR1B	1.45665	8.12E-14	1.8E-13	7.956416	Up	arginine vasopressin receptor 1B
SMCR8	1.454114	1.48E-30	3.64E-28	13.38103	Up	SMCR8-C9orf72 complex subunit
ELK1	1.451398	5.47E-23	2.13E-22	11.03648	Up	ETS transcription factor ELK1
RAI1	1.450765	7.47E-29	1.49E-26	12.86049	Up	retinoic acid induced 1
MIGA2	1.450256	9.85E-46	4.78E-42	17.99928	Up	mitoguardin 2
SEC16A	1.449629	2.17E-45	9.04E-42	17.89343	Up	SEC16 homolog A, endoplasmic reticulum export factor
TMSB4Y	1.448197	3.63E-23	1.44E-22	11.09291	Up	thymosin beta 4 Y-linked
KCNG3	1.447929	1.22E-16	3.08E-16	8.952739	Up	potassium voltage-gated channel modifier subfamily G member 3
KLHL15	1.441575	1.69E-27	2.74E-25	12.44393	Up	kelch like family member 15
SLC37A2	1.439392	1.19E-24	5E-23	11.56003	Up	solute carrier family 37 member 2
PDE4A	1.438872	3.84E-16	9.49E-16	8.780866	Up	phosphodiesterase 4A
CELA3B	1.437677	2.09E-13	4.55E-13	7.806062	Up	chymotrypsin like elastase 3B
TUBB3	1.437504	1.88E-11	3.74E-11	7.067846	Up	tubulin beta 3 class III
GVINP1	1.435197	6.52E-17	1.67E-16	9.045562	Up	GTPase, very large interferon inducible pseudogene 1
PI4KA	1.431833	1.83E-41	2.48E-38	16.69225	Up	phosphatidylinositol 4-kinase alpha
RNF123	1.431003	1.58E-42	2.79E-39	17.01646	Up	ring finger protein 123
CMTR1	1.430304	4.68E-40	5.04E-37	16.26397	Up	cap methyltransferase 1
FGFR3	1.430102	4.96E-44	1.37E-40	17.4766	Up	fibroblast growth factor receptor 3
SEMA6C	1.422171	1.25E-29	2.66E-27	13.09773	Up	semaphorin 6C
NLGN4Y	1.419677	3.34E-21	1.12E-20	10.46588	Up	neuroligin 4 Y-linked
CUL7	1.414311	1.16E-28	2.24E-26	12.80171	Up	cullin 7
ITGB4	1.414184	8.75E-42	1.27E-38	16.78991	Up	integrin subunit beta 4
SYT7	1.413869	2.42E-23	9.94E-23	11.1487	Up	synaptotagmin 7
OAS3	1.409483	4.93E-19	1.46E-18	9.75804	Up	2'-5'-oligoadenylate synthetase 3
CDON	1.408012	3.81E-15	8.98E-15	8.432346	Up	cell adhesion associated, oncogene regulated
PTPRB	1.403807	2.37E-27	3.75E-25	12.39913	Up	protein tyrosine phosphatase receptor type B
ATRNL1	1.398476	6.06E-12	1.23E-11	7.257681	Up	attractin like 1

CASKIN2	1.398006	1.02E-34	4.33E-32	14.64527	Up	CASK interacting protein 2
BSN	1.395314	1.49E-11	2.97E-11	7.107318	Up	bassoon presynaptic cytomatrix protein
KHNYN	1.391888	2.17E-41	2.68E-38	16.66992	Up	KH and NYN domain containing
BRPF1	1.388276	2.87E-43	6.41E-40	17.24323	Up	bromodomain and PHD finger containing 1
SLC26A1	1.38292	2.68E-25	3.09E-23	11.76257	Up	solute carrier family 26 member 1
MAPKBP1	1.381821	2.32E-34	9.32E-32	14.53685	Up	mitogen-activated protein kinase binding protein 1
TRIM3	1.380962	3.23E-31	8.57E-29	13.58257	Up	tripartite motif containing 3
ATXN2L	1.379909	1.94E-38	1.41E-35	15.77285	Up	ataxin 2 like
TOR4A	1.376873	1.85E-28	3.49E-26	12.73946	Up	torsin family 4 member A
TMEM201	1.376452	1.18E-12	2.48E-12	7.527023	Up	transmembrane protein 201
AKR1B15	1.373679	1.46E-07	2.41E-07	5.427009	Up	aldo-ketoreductase family 1 member B15
KLHL21	1.372247	1.16E-30	2.91E-28	13.41373	Up	kelch like family member 21
POFUT2	1.37097	7.06E-34	2.63E-31	14.39044	Up	protein O-fucosyltransferase 2
RPS6KA4	1.370687	2.61E-24	5E-23	11.45342	Up	ribosomal protein S6 kinase A4
RTEL1-TNFRSF6B	1.370426	1.42E-17	3.72E-17	9.27079	Up	RTEL1-TNFRSF6B readthrough (NMD candidate)
ADGRB2	1.368096	1.98E-41	2.5E-38	16.682	Up	adhesion G protein-coupled receptor B2
MED12	1.366559	3.09E-40	3.46E-37	16.31865	Up	mediator complex subunit 12
MAPK4	1.362743	6.23E-11	1.21E-10	6.863409	Up	mitogen-activated protein kinase 4
EVI5L	1.362231	2.59E-27	4.07E-25	12.38697	Up	ecotropic viral integration site 5 like
DOCK10	1.362067	6.57E-23	2.53E-22	11.01115	Up	dedicator of cytokinesis 10
ACAP3	1.360748	5.31E-28	9.28E-26	12.59892	Up	ArfGAP with coiled-coil, ankyrin repeat and PH domains 3
CLUH	1.359859	2.39E-32	7.27E-30	13.92641	Up	clustered mitochondria homolog
RAVER1	1.355045	1.24E-38	9.26E-36	15.83164	Up	ribonucleoprotein, PTB binding 1
PLEC	1.353963	1.52E-33	5.42E-31	14.28969	Up	plectin
NPC1	1.350595	3.21E-43	6.91E-40	17.22841	Up	NPC intracellular cholesterol transporter 1
TNS2	1.346251	1.36E-19	4.26E-19	9.941869	Up	tensin 2
B4GALNT3	1.343671	5.57E-11	1.08E-10	6.88282	Up	beta-1,4-N-acetyl-galactosaminyltransferase 3
ARHGAP27	1.340067	6.96E-40	6.98E-37	16.2115	Up	Rho GTPase activating protein 27
SEMA3D	1.338877	1.76E-09	3.2E-09	6.272075	Up	semaphorin 3D
MYO15B	1.33787	1.55E-35	7.47E-33	14.89275	Up	myosin XVB
PLEKHH1	1.337471	3.6E-38	2.49E-35	15.69134	Up	pleckstrin homology, MyTH4 and FERM domain containing H1
SNTB1	1.336176	1.44E-17	3.8E-17	9.267783	Up	syntrophin beta 1
VLDLR	1.33577	1.28E-10	2.46E-10	6.73873	Up	very low density lipoprotein receptor

ARHGEF18	1.334008	1.65E-20	5.37E-20	10.24134	Up	Rho/Rac guanine nucleotide exchange factor 18
CYP2B6	1.332978	3.34E-10	6.29E-10	6.570419	Up	cytochrome P450 family 2 subfamily B member 6
CUL9	1.332622	6.28E-35	2.77E-32	14.70907	Up	cullin 9
LRP4	1.330979	1.15E-24	5E-23	11.56532	Up	LDL receptor related protein 4
ALS2CL	1.326535	1.17E-30	2.93E-28	13.41248	Up	ALS2 C-terminal like
TMEM132C	1.324259	1.91E-09	3.47E-09	6.257252	Up	transmembrane protein 132C
GRINA	1.323974	1.46E-32	4.56E-30	13.99161	Up	glutamate ionotropic receptor NMDA type subunit associated protein 1
IGSF9	1.323933	6.45E-27	9.64E-25	12.26479	Up	immunoglobulin superfamily member 9
LDLR	1.322432	1.41E-23	5.99E-23	11.22282	Up	low density lipoprotein receptor
FYCO1	1.320725	1.1E-29	2.35E-27	13.11509	Up	FYVE and coiled-coil domain containing 1
SLC25A25	1.318164	3.2E-29	6.63E-27	12.97304	Up	solute carrier family 25 member 25
MAP11	1.316368	5.3E-27	8.04E-25	12.29117	Up	microtubule associated protein 11
SHROOM3	1.316237	1.96E-39	1.87E-36	16.07489	Up	shroom family member 3
ZKSCAN8	1.315725	6.79E-18	1.82E-17	9.378237	Up	zinc finger with KRAB and SCAN domains 8
NDST1	1.315386	6.63E-33	2.12E-30	14.09536	Up	N-deacetylase and N-sulfotransferase 1
SARM1	1.313233	7.17E-22	2.5E-21	10.68046	Up	sterile alpha and TIR motif containing 1
POLG	1.313108	1.08E-40	1.24E-37	16.45712	Up	DNA polymerase gamma, catalytic subunit
ADAM15	1.310967	3.23E-39	2.8E-36	16.00907	Up	ADAM metallopeptidase domain 15
MYORG	1.30949	1.55E-28	2.94E-26	12.76346	Up	myogenesis regulating glycosidase (putative)
PKP3	1.309219	2.82E-39	2.56E-36	16.02694	Up	plakophilin 3
SLC6A19	1.308961	6.18E-35	2.74E-32	14.7112	Up	solute carrier family 6 member 19
PLXNB2	1.308813	3.42E-43	7.1E-40	17.21986	Up	plexin B2
PSD4	1.308678	7.02E-36	3.44E-33	14.99738	Up	pleckstrin and Sec7 domain containing 4
GRK2	1.30789	2.8E-33	9.65E-31	14.20891	Up	G protein-coupled receptor kinase 2
PCSK9	1.305073	7.2E-11	1.4E-10	6.83845	Up	proproteinconvertasesubtilisin/kexin type 9
SLC22A23	1.304428	1.31E-32	4.12E-30	14.00555	Up	solute carrier family 22 member 23
HIC1	1.304188	4.21E-16	1.04E-15	8.766818	Up	HIC ZBTB transcriptional repressor 1
MAP3K14	1.303126	6.8E-28	1.18E-25	12.56597	Up	mitogen-activated protein kinase kinasekinase 14
EPS8L2	1.300391	8.2E-39	6.44E-36	15.88634	Up	EPS8 like 2
TYK2	1.300207	3.37E-40	3.69E-37	16.30743	Up	tyrosine kinase 2
DPP10	1.299161	8.87E-11	1.71E-10	6.802442	Up	dipeptidyl peptidase like 10
PLXNB1	1.298898	2.73E-33	9.46E-31	14.21223	Up	plexin B1
IQGAP3	1.296364	3.44E-10	6.46E-10	6.565309	Up	IQ motif containing GTPase activating protein 3
CAMSAP3	1.293495	6.11E-40	6.43E-37	16.22871	Up	calmodulin regulated spectrin associated protein family member 3

SFMBT2	1.292954	4.62E-15	1.08E-14	8.40284	Up	Scm like with four mbt domains 2
ABCA2	1.292794	2.95E-23	1.18E-22	11.12119	Up	ATP binding cassette subfamily A member 2
LZTR1	1.29243	2.62E-31	7.05E-29	13.61013	Up	leucine zipper like transcription regulator 1
CACNA1C	1.291878	6.62E-13	1.41E-12	7.621104	Up	calcium voltage-gated channel subunit alpha1 C
VAV2	1.288686	4.62E-33	1.52E-30	14.14292	Up	vav guanine nucleotide exchange factor 2
SHC1	1.288501	3.2E-34	1.27E-31	14.49473	Up	SHC adaptor protein 1
RNF40	1.287602	1.46E-35	7.09E-33	14.90081	Up	ring finger protein 40
ENAM	1.286926	1.82E-13	3.97E-13	7.828002	Up	enamelin
PCDH1	1.286291	7.85E-35	3.43E-32	14.67964	Up	protocadherin 1
PLXNA3	1.286207	6.04E-25	5E-23	11.65219	Up	plexin A3
ITPKB	1.282803	4.16E-25	4.57E-23	11.70265	Up	inositol-trisphosphate 3-kinase B
RALGDS	1.281353	7.51E-34	2.78E-31	14.3824	Up	ral guanine nucleotide dissociation stimulator
PHKA1	1.281196	4.77E-14	1.07E-13	8.040361	Up	phosphorylase kinase regulatory subunit alpha 1
JAG1	1.275976	2.71E-39	2.51E-36	16.03208	Up	jagged canonical Notch ligand 1
CIITA	1.27375	3.25E-35	1.49E-32	14.79576	Up	class II major histocompatibility complex transactivator
MROH1	1.27353	1.25E-36	6.89E-34	15.22389	Up	maestro heat like repeat family member 1
ARHGAP19	1.269088	1.8E-23	7.62E-23	11.18914	Up	Rho GTPase activating protein 19
OSBPL7	1.267914	4.47E-39	3.66E-36	15.96633	Up	oxysterol binding protein like 7
ZDHHC11	1.267333	3.37E-09	6.06E-09	6.152463	Up	zinc finger DHHC-type containing 11
MPRIP	1.265858	5E-32	1.46E-29	13.82879	Up	myosin phosphatase Rho interacting protein
PASK	1.264991	2.2E-21	7.47E-21	10.52388	Up	PAS domain containing serine/threonine kinase
STK36	1.264879	5.2E-26	6.91E-24	11.98393	Up	serine/threonine kinase 36
PFKFB3	1.262301	4.44E-24	5E-23	11.3807	Up	6-phosphofructo-2-kinase/fructose-2,6-biphosphatase 3
GPR153	1.26165	4.33E-25	4.72E-23	11.69751	Up	G protein-coupled receptor 153
HAS3	1.261534	6.28E-12	1.28E-11	7.251728	Up	hyaluronan synthase 3
LAMB2	1.261435	4.05E-32	1.19E-29	13.85672	Up	laminin subunit beta 2
DMTN	1.261079	1.27E-17	3.35E-17	9.286461	Up	dematin actin binding protein
MYO1D	1.259221	7.03E-36	3.44E-33	14.99716	Up	myosin ID
FZD1	1.259099	1.11E-27	1.86E-25	12.50084	Up	frizzled class receptor 1
ELF4	1.258154	1.47E-30	3.64E-28	13.38196	Up	E74 like ETS transcription factor 4
MYBBP1A	1.25632	8.84E-22	3.06E-21	10.65133	Up	MYB binding protein 1a
ZNF710	1.254845	2.75E-19	8.34E-19	9.841749	Up	zinc finger protein 710
TNKS1BP1	1.254473	1.13E-27	1.89E-25	12.49852	Up	tankyrase 1 binding protein 1
CARNS1	1.253973	7.75E-12	1.57E-11	7.216839	Up	carnosine synthase 1

LAMC1	1.252621	1.45E-09	2.65E-09	6.307249	Up	laminin subunit gamma 1
SNX33	1.251871	2.14E-30	5.11E-28	13.33198	Up	sorting nexin 33
ZNF592	1.24988	5.51E-36	2.74E-33	15.02929	Up	zinc finger protein 592
SBNO2	1.249577	6.84E-32	1.96E-29	13.7875	Up	strawberry notch homolog 2
CGN	1.247998	1.76E-35	8.38E-33	14.8766	Up	cingulin
ZNF653	1.245497	1.36E-16	3.44E-16	8.936023	Up	zinc finger protein 653
SSTR1	1.245492	1.11E-26	1.6E-24	12.19184	Up	somatostatin receptor 1
CDAN1	1.24513	4.83E-33	1.58E-30	14.13706	Up	codanin 1
NUP188	1.241392	1.62E-27	2.63E-25	12.45005	Up	nucleoporin 188
ADAMTS14	1.240863	4.13E-09	7.39E-09	6.114986	Up	ADAM metallopeptidase with thrombospondin type 1 motif 14
TMEM94	1.240715	7.06E-37	4.23E-34	15.29966	Up	transmembrane protein 94
MVB12B	1.240662	8.29E-22	2.88E-21	10.66026	Up	multivesicular body subunit 12B
PTK7	1.239845	1.52E-10	2.9E-10	6.709082	Up	protein tyrosine kinase 7 (inactive)
LDHAL6B	1.239622	6.55E-13	1.39E-12	7.622654	Up	lactate dehydrogenase A like 6B
KL	1.239394	3.36E-17	8.69E-17	9.143759	Up	klotho
ZBTB7C	1.237497	1.65E-13	3.59E-13	7.844345	Up	zinc finger and BTB domain containing 7C
SPECC1L	1.237399	1.5E-20	4.91E-20	10.25428	Up	sperm antigen with calponin homology and coiled-coil domains 1 like
IRGQ	1.234884	5.33E-20	1.7E-19	10.07556	Up	immunity related GTPase Q
ADM2	1.233725	2.77E-19	8.4E-19	9.840727	Up	adrenomedullin 2
WNT9B	1.23253	3.44E-12	7.06E-12	7.351847	Up	Wnt family member 9B
ANK1	1.232382	2E-12	4.15E-12	7.441065	Up	ankyrin 1
SCRN1	1.232101	4.85E-11	9.47E-11	6.90643	Up	secernin 1
TFE3	1.228791	1.77E-36	9.55E-34	15.17846	Up	transcription factor binding to IGHM enhancer 3
PRPF8	1.227812	1.12E-36	6.24E-34	15.23939	Up	pre-mRNA processing factor 8
KLHL25	1.227723	8.09E-29	1.6E-26	12.84985	Up	kelch like family member 25
SV2B	1.227027	8.37E-09	1.48E-08	5.982765	Up	synaptic vesicle glycoprotein 2B
ZNF853	1.226838	3.27E-11	6.43E-11	6.973936	Up	zinc finger protein 853
CHRNB2	1.22677	4.19E-11	8.19E-11	6.931658	Up	cholinergic receptor nicotinic beta 2 subunit
USP19	1.226667	8.2E-38	5.42E-35	15.58298	Up	ubiquitin specific peptidase 19
ADCY3	1.225283	6.73E-12	1.37E-11	7.240358	Up	adenylatecyclase 3
SNAPC4	1.223639	1.14E-27	1.9E-25	12.49725	Up	small nuclear RNA activating complex polypeptide 4
HDAC6	1.22202	3.15E-35	1.47E-32	14.7999	Up	histone deacetylase 6
MALRD1	1.221652	6.42E-47	4.15E-43	18.36519	Up	MAM and LDL receptor class A domain containing 1
TRAF7	1.221208	3.26E-35	1.49E-32	14.79549	Up	TNF receptor associated factor 7

PARP10	1.219068	1.24E-34	5.13E-32	14.62	Up	poly(ADP-ribose) polymerase family member 10
NOS2	1.219044	2.52E-11	4.98E-11	7.018494	Up	nitric oxide synthase 2
COL17A1	1.218573	2.25E-22	8.22E-22	10.84112	Up	collagen type XVII alpha 1 chain
MEGF6	1.218567	1.46E-12	3.05E-12	7.49266	Up	multiple EGF like domains 6
FASN	1.218082	4.97E-16	1.22E-15	8.741984	Up	fatty acid synthase
CAD	1.217215	1.87E-21	6.36E-21	10.54678	Up	carbamoyl-phosphate synthetase 2, aspartate transcarbamylase, and dihydroorotase
SREBF1	1.217108	2.09E-28	3.86E-26	12.72331	Up	sterol regulatory element binding transcription factor 1
CHRNA2	1.216726	1.36E-09	2.49E-09	6.319215	Up	cholinergic receptor nicotinic alpha 2 subunit
PIP5K1C	1.216458	1.21E-30	3.02E-28	13.40768	Up	phosphatidylinositol-4-phosphate 5-kinase type 1 gamma
PLD2	1.215839	3.07E-23	1.23E-22	11.11572	Up	phospholipase D2
IGSF10	1.215559	1.5E-09	2.74E-09	6.300958	Up	immunoglobulin superfamily member 10
KCNK2	1.215082	1.68E-09	3.07E-09	6.280347	Up	potassium two pore domain channel subfamily K member 2
NOTCH1	1.212654	9.38E-22	3.24E-21	10.64307	Up	notch receptor 1
ATP8B2	1.21031	3.17E-23	1.27E-22	11.1114	Up	ATPase phospholipid transporting 8B2
MCM3AP	1.210172	9.31E-34	3.4E-31	14.35414	Up	minichromosome maintenance complex component 3 associated protein
CARD14	1.208591	1.09E-10	2.1E-10	6.766595	Up	caspase recruitment domain family member 14
CC2D1B	1.208178	5.83E-30	1.33E-27	13.19932	Up	coiled-coil and C2 domain containing 1B
SH3TC1	1.20809	9.65E-15	2.22E-14	8.289424	Up	SH3 domain and tetratricopeptide repeats 1
DMWD	1.206894	9.8E-26	1.25E-23	11.89841	Up	DM1 locus, WD repeat containing
UPF1	1.206296	1.98E-38	1.42E-35	15.7701	Up	UPF1 RNA helicase and ATPase
ARHGEF7	1.205138	5.5E-37	3.37E-34	15.33235	Up	Rho guanine nucleotide exchange factor 7
PML	1.204509	4.03E-37	2.52E-34	15.37338	Up	promyelocyticleukemia
NEURL4	1.204114	1.54E-28	2.93E-26	12.76422	Up	neuralized E3 ubiquitin protein ligase 4
PRDM15	1.203758	1.09E-17	2.89E-17	9.309028	Up	PR/SET domain 15
TRPM4	1.20322	3.36E-42	5.58E-39	16.91687	Up	transient receptor potential cation channel subfamily M member 4
ADAMTS13	1.203122	2.39E-28	4.3E-26	12.70564	Up	ADAM metallopeptidase with thrombospondin type 1 motif 13
FRMD8	1.202153	4.97E-27	7.61E-25	12.29971	Up	FERM domain containing 8
TAOK2	1.201121	1.64E-41	2.27E-38	16.7069	Up	TAO kinase 2
ITGA3	1.201072	9.94E-38	6.43E-35	15.55758	Up	integrin subunit alpha 3
PTPRH	1.200925	2.84E-36	1.49E-33	15.11657	Up	protein tyrosine phosphatase receptor type H
ZNF335	1.200858	4.1E-32	1.2E-29	13.8552	Up	zinc finger protein 335
ERBB2	1.200799	2.47E-44	7.56E-41	17.56945	Up	erb-b2 receptor tyrosine kinase 2
RAPGEF4	1.200596	2.87E-14	6.47E-14	8.120065	Up	Rap guanine nucleotide exchange factor 4
SCARF2	1.199636	1.29E-15	3.12E-15	8.59762	Up	scavenger receptor class F member 2

INPP5E	1.196891	1.47E-31	4.04E-29	13.68623	Up	inositol polyphosphate-5-phosphatase E
SETD7	1.196875	1.47E-10	2.81E-10	6.714661	Up	SET domain containing 7, histone lysine methyltransferase
CNOT3	1.196802	3.95E-35	1.78E-32	14.77014	Up	CCR4-NOT transcription complex subunit 3
GMIP	1.196017	1.5E-34	6.17E-32	14.594	Up	GEM interacting protein
CDC42EP4	1.195596	3.39E-32	1E-29	13.88009	Up	CDC42 effector protein 4
INAVA	1.19543	7.04E-42	1.05E-38	16.81878	Up	innate immunity activator
MAP3K10	1.195339	5.38E-15	1.26E-14	8.37957	Up	mitogen-activated protein kinase kinasekinase 10
DENND4B	1.195072	9.51E-32	2.7E-29	13.74406	Up	DENN domain containing 4B
TMEM63B	1.192941	1.01E-31	2.86E-29	13.73568	Up	transmembrane protein 63B
SLC4A2	1.192654	2.36E-26	3.28E-24	12.09012	Up	solute carrier family 4 member 2
ATP9A	1.191902	3.7E-31	9.6E-29	13.56461	Up	ATPase phospholipid transporting 9A (putative)
PLPPR5	1.188892	2.65E-09	4.8E-09	6.196512	Up	phospholipid phosphatase related 5
MYO7A	1.187845	4.05E-15	9.52E-15	8.42328	Up	myosin VIIA
DNHD1	1.187333	2.02E-23	8.36E-23	11.17312	Up	dynein heavy chain domain 1
BTBD11	1.186279	1.03E-13	2.27E-13	7.918826	Up	BTB domain containing 11
SMURF1	1.184298	2.11E-34	8.54E-32	14.54934	Up	SMAD specific E3 ubiquitin protein ligase 1
EPAS1	1.184006	2.93E-33	1E-30	14.20314	Up	endothelial PAS domain protein 1
DACT1	1.183651	2.97E-09	5.35E-09	6.176045	Up	dishevelled binding antagonist of beta catenin 1
PPM1E	1.182509	6.78E-11	1.32E-10	6.848862	Up	protein phosphatase, Mg2+/Mn2+ dependent 1E
SKIV2L	1.181866	1.25E-27	2.08E-25	12.48445	Up	Ski2 like RNA helicase
SNRNP200	1.180972	3.12E-39	2.77E-36	16.01385	Up	small nuclear ribonucleoprotein U5 subunit 200
PLXNA2	1.180269	1.8E-43	4.37E-40	17.30499	Up	plexin A2
ADGRG2	1.179694	1.83E-08	3.17E-08	5.83473	Up	adhesion G protein-coupled receptor G2
CDC42BPG	1.178683	1.81E-33	6.41E-31	14.26679	Up	CDC42 binding protein kinase gamma
PNPLA6	1.178373	6.56E-26	8.59E-24	11.95262	Up	patatin like phospholipase domain containing 6
HYOU1	1.177352	5.06E-20	1.61E-19	10.08278	Up	hypoxia up-regulated 1
NATD1	1.176898	5.21E-17	1.34E-16	9.07884	Up	N-acetyltransferase domain containing 1
LOC100507403	1.175074	1.08E-11	2.17E-11	7.161211	Up	uncharacterized LOC100507403
POLRMT	1.173917	2.15E-36	1.15E-33	15.15308	Up	RNA polymerase mitochondrial
ZC3H3	1.17348	6.31E-32	1.82E-29	13.79819	Up	zinc finger CCCH-type containing 3
CCDC88B	1.172998	8.24E-35	3.58E-32	14.67326	Up	coiled-coil domain containing 88B
MAST2	1.172945	2.37E-35	1.12E-32	14.83754	Up	microtubule associated serine/threonine kinase 2
PRAG1	1.172612	3.74E-28	6.61E-26	12.64582	Up	PEAK1 related, kinase-activating pseudokinase 1
TBC1D9B	1.171279	3.44E-34	1.32E-31	14.48536	Up	TBC1 domain family member 9B

CIC	1.171059	3.85E-21	1.29E-20	10.4458	Up	capicua transcriptional repressor
UNC13B	1.170932	8.18E-23	3.1E-22	10.98097	Up	unc-13 homolog B
FAM83E	1.169242	3.27E-39	2.8E-36	16.00729	Up	family with sequence similarity 83 member E
AP1B1	1.16866	3.25E-34	1.27E-31	14.49262	Up	adaptor related protein complex 1 subunit beta 1
PLXNA4	1.168473	2.22E-10	4.21E-10	6.642336	Up	plexin A4
RAP1GAP2	1.168419	4.44E-16	1.09E-15	8.759045	Up	RAP1 GTPase activating protein 2
CTNND1	1.167602	8.91E-39	6.91E-36	15.87537	Up	catenin delta 1
TTC7A	1.166221	3.69E-31	9.6E-29	13.5649	Up	tetratricopeptide repeat domain 7A
TIAM2	1.16592	4.14E-43	8.3E-40	17.19444	Up	TIAM Rac1 associated GEF 2
ADCY6	1.165055	6.76E-27	1.01E-24	12.25844	Up	adenylatecyclase 6
ZNF585B	1.16487	2.09E-25	2.51E-23	11.79634	Up	zinc finger protein 585B
HDAC5	1.163942	7.23E-37	4.25E-34	15.29643	Up	histone deacetylase 5
ZAP70	1.163763	6.69E-15	1.56E-14	8.345948	Up	zeta chain of T cell receptor associated protein kinase 70
PER3	1.162951	1.5E-18	4.24E-18	9.597729	Up	period circadian regulator 3
TMEM132A	1.161006	4.78E-10	8.94E-10	6.50666	Up	transmembrane protein 132A
ABCA7	1.160993	4.76E-21	1.59E-20	10.41605	Up	ATP binding cassette subfamily A member 7
MINK1	1.157817	3.25E-30	7.6E-28	13.27675	Up	misshapen like kinase 1
FAAP100	1.157425	6.9E-30	1.54E-27	13.17689	Up	FA core complex associated protein 100
LINC00639	1.156611	9.92E-09	1.75E-08	5.950806	Up	long intergenic non-protein coding RNA 639
SH3RF3	1.155606	1.62E-13	3.54E-13	7.846718	Up	SH3 domain containing ring finger 3
EDEM1	1.155075	7.27E-30	1.61E-27	13.17012	Up	ER degradation enhancing alpha-mannosidase like protein 1
CIT	1.154649	2.72E-10	5.13E-10	6.606877	Up	citron rho-interacting serine/threonine kinase
GPR55	1.154308	3.3E-11	6.5E-11	6.972117	Up	G protein-coupled receptor 55
DOCK2	1.152764	3.42E-16	8.49E-16	8.798006	Up	dedicator of cytokinesis 2
CSRNP1	1.152225	3.56E-30	8.29E-28	13.26471	Up	cysteine and serine rich nuclear protein 1
IP6K1	1.151613	3.95E-35	1.78E-32	14.76992	Up	inositol hexakisphosphate kinase 1
SUN2	1.150709	3.08E-33	1.05E-30	14.1965	Up	Sad1 and UNC84 domain containing 2
TRPM2	1.150629	1.58E-12	3.3E-12	7.479388	Up	transient receptor potential cation channel subfamily M member 2
NRBP2	1.150419	1.45E-24	5E-23	11.53317	Up	nuclear receptor binding protein 2
TEF	1.149278	3.54E-17	9.15E-17	9.135943	Up	TEF transcription factor, PAR bZIP family member
ANKFY1	1.148282	3.67E-31	9.6E-29	13.56565	Up	ankyrin repeat and FYVE domain containing 1
FOSL2	1.147624	1.12E-33	4.04E-31	14.33012	Up	FOS like 2, AP-1 transcription factor subunit
FOXJ2	1.147008	1.5E-29	3.17E-27	13.07353	Up	forkhead box J2
SNX30	1.14559	3.57E-33	1.2E-30	14.17714	Up	sorting nexin family member 30

MYRF	1.14547	6.07E-29	1.22E-26	12.88815	Up	myelin regulatory factor
ENGASE	1.145247	2.41E-25	2.83E-23	11.77649	Up	endo-beta-N-acetylglucosaminidase
YBX2	1.144879	3.61E-23	1.44E-22	11.09367	Up	Y-box binding protein 2
CHRM4	1.143298	6.6E-10	1.23E-09	6.44916	Up	cholinergic receptor muscarinic 4
PLEKHG1	1.142759	1.23E-21	4.21E-21	10.60585	Up	pleckstrin homology and RhoGEF domain containing G1
TCF23	1.142327	3.11E-11	6.13E-11	6.982164	Up	transcription factor 23
CPD	1.142088	3.07E-32	9.15E-30	13.89343	Up	carboxypeptidase D
EPHA10	1.141639	3.24E-12	6.67E-12	7.361431	Up	EPH receptor A10
CASR	1.141636	3.57E-09	6.42E-09	6.141708	Up	calcium sensing receptor
SLC6A6	1.140323	2.48E-30	5.88E-28	13.31289	Up	solute carrier family 6 member 6
LOC100507516	1.140264	5.2E-13	1.11E-12	7.659922	Up	uncharacterized LOC100507516
CPLX2	1.139239	1.7E-07	2.8E-07	5.396063	Up	complexin 2
DISP3	1.138523	4.67E-09	8.33E-09	6.092243	Up	dispatched RND transporter family member 3
GBF1	1.138152	1.02E-39	1.01E-36	16.16093	Up	golgibrefeldin A resistant guanine nucleotide exchange factor 1
RASAL1	1.137939	2.2E-13	4.77E-13	7.798147	Up	RAS protein activator like 1
SLC30A1	1.137522	1.24E-39	1.2E-36	16.13535	Up	solute carrier family 30 member 1
CDK18	1.136852	1.42E-18	4.02E-18	9.605942	Up	cyclin dependent kinase 18
MARCHF4	1.136531	5.61E-10	1.05E-09	6.478141	Up	membrane associated ring-CH-type finger 4
TCF3	1.135875	1.47E-27	2.41E-25	12.46284	Up	transcription factor 3
CLTC	1.135875	1.87E-30	4.49E-28	13.35016	Up	clathrin heavy chain
PSMD6-AS2	1.135025	1.75E-14	3.98E-14	8.197224	Up	PSMD6 antisense RNA 2
DGKD	1.134418	6.7E-29	1.34E-26	12.87488	Up	diacylglycerol kinase delta
CAND2	1.134332	1.54E-09	2.81E-09	6.296393	Up	cullin associated and neddylation dissociated 2 (putative)
GSTM1	1.134331	0.000525	0.000708	3.51758	Up	glutathione S-transferase mu 1
CCDC120	1.133708	1.18E-24	5E-23	11.56179	Up	coiled-coil domain containing 120
U2AF1L5	1.132326	1.97E-10	3.74E-10	6.663731	Up	U2 small nuclear RNA auxiliary factor 1 like 5
SNX19	1.131879	2.04E-29	4.26E-27	13.03319	Up	sorting nexin 19
KRT73-AS1	1.131867	1.13E-11	2.27E-11	7.153696	Up	KRT73 antisense RNA 1
CLCN7	1.131542	6.21E-32	1.8E-29	13.80037	Up	chloride voltage-gated channel 7
PDE3A	1.131007	4.95E-19	1.46E-18	9.757323	Up	phosphodiesterase 3A
TEN1-CDK3	1.130859	9.45E-13	1.99E-12	7.56331	Up	TEN1-CDK3 readthrough (NMD candidate)
TOM1L2	1.129347	5.26E-31	1.35E-28	13.51803	Up	target of myb1 like 2 membrane trafficking protein
FAM166A	1.128474	1.27E-17	3.36E-17	9.286335	Up	family with sequence similarity 166 member A
KIFC3	1.128205	1.52E-21	5.19E-21	10.57592	Up	kinesin family member C3

PLA2G4F	1.128166	2.88E-31	7.69E-29	13.5976	Up	phospholipase A2 group IVF
PPL	1.127921	2.33E-20	7.54E-20	10.19262	Up	periplakin
LAMA5	1.127847	2.33E-10	4.41E-10	6.634016	Up	laminin subunit alpha 5
NUP205	1.126368	8.45E-25	5E-23	11.60668	Up	nucleoporin 205
FBXO41	1.125839	3.66E-10	6.87E-10	6.554331	Up	F-box protein 41
NDOR1	1.125304	1.02E-27	1.73E-25	12.512	Up	NADPH dependent diflavinoxidoreductase 1
LMF2	1.125202	4.1E-36	2.09E-33	15.06812	Up	lipase maturation factor 2
NLRP6	1.12352	9.07E-29	1.78E-26	12.83468	Up	NLR family pyrin domain containing 6
GPT	1.123027	6.61E-30	1.48E-27	13.18266	Up	glutamic--pyruvic transaminase
FZR1	1.122309	3.64E-31	9.59E-29	13.56645	Up	fizzy and cell division cycle 20 related 1
RAB35	1.121888	3.76E-21	1.26E-20	10.4493	Up	RAB35, member RAS oncogene family
PDZD7	1.121638	1.93E-22	7.08E-22	10.86244	Up	PDZ domain containing 7
SLC25A22	1.121487	5.12E-22	1.8E-21	10.72722	Up	solute carrier family 25 member 22
ARID3B	1.121461	9.19E-30	1.99E-27	13.13899	Up	AT-rich interaction domain 3B
DCTN1	1.120671	7.16E-31	1.83E-28	13.47717	Up	dynactin subunit 1
ATP13A3	1.12016	6.69E-25	5E-23	11.63834	Up	ATPase 13A3
PLEKHN1	1.120105	3.41E-11	6.7E-11	6.96665	Up	pleckstrin homology domain containing N1
WDR90	1.119524	3.94E-24	5E-23	11.39697	Up	WD repeat domain 90
ZBTB40	1.118796	1.83E-25	2.23E-23	11.81398	Up	zinc finger and BTB domain containing 40
DOCK9	1.118672	8.55E-27	1.26E-24	12.22688	Up	dedicator of cytokinesis 9
PAN2	1.117687	2.34E-28	4.24E-26	12.7085	Up	poly(A) specific ribonuclease subunit PAN2
SEC24C	1.117384	1.13E-29	2.42E-27	13.11093	Up	SEC24 homolog C, COPII coat complex component
CDHR2	1.117294	1.12E-30	2.84E-28	13.41775	Up	cadherin related family member 2
PLEKHM1	1.117187	1.76E-32	5.4E-30	13.96644	Up	pleckstrin homology and RUN domain containing M1
CAMTA2	1.115108	1.42E-27	2.35E-25	12.46718	Up	calmodulin binding transcription activator 2
LRP5	1.115084	5.9E-30	1.34E-27	13.1978	Up	LDL receptor related protein 5
ARNT2	1.114843	6.02E-12	1.22E-11	7.25892	Up	aryl hydrocarbon receptor nuclear translocator 2
CLSTN3	1.114691	1.92E-17	5.01E-17	9.226366	Up	calsyntenin 3
LINGO1	1.11432	6.24E-12	1.27E-11	7.252917	Up	leucine rich repeat and Ig domain containing 1
FCHSD1	1.113723	1.06E-20	3.47E-20	10.30395	Up	FCH and double SH3 domains 1
AGRN	1.113599	1.67E-22	6.13E-22	10.88277	Up	agrin
PIK3AP1	1.11299	2.45E-12	5.07E-12	7.407609	Up	phosphoinositide-3-kinase adaptor protein 1
SPTBN1	1.112327	2.11E-31	5.78E-29	13.63843	Up	spectrin beta, non-erythrocytic 1
PLEKHG4	1.11213	2.22E-12	4.6E-12	7.423975	Up	pleckstrin homology and RhoGEF domain containing G4

IFI44L	1.112023	3.64E-06	5.59E-06	4.747136	Up	interferon induced protein 44 like
XIST	-3.57042	3.52E-15	8.31E-15	-8.44462	Down	X inactive specific transcript
S100A9	-2.65966	9.95E-14	2.19E-13	-7.92433	Down	S100 calcium binding protein A9
MNDA	-2.49959	4.16E-11	8.14E-11	-6.93277	Down	myeloid cell nuclear differentiation antigen
SELL	-2.44498	1.63E-11	3.25E-11	-7.09215	Down	selectin L
FPR1	-2.42044	2.81E-11	5.55E-11	-6.99963	Down	formyl peptide receptor 1
S100A8	-2.30177	3.64E-11	7.14E-11	-6.95557	Down	S100 calcium binding protein A8
IL1R2	-2.18286	2.43E-19	7.45E-19	-9.85929	Down	interleukin 1 receptor type 2
FCGR3B	-2.16931	2.73E-10	5.15E-10	-6.6063	Down	Fc fragment of IgG receptor IIIb
CLEC4E	-2.14996	6.04E-15	1.41E-14	-8.36178	Down	C-type lectin domain family 4 member E
SPINK5	-2.08897	6.86E-08	1.16E-07	-5.57709	Down	serine peptidase inhibitor, Kazal type 5
CXCR1	-2.06326	2.56E-12	5.29E-12	-7.40053	Down	C-X-C motif chemokine receptor 1
S100A12	-1.95157	3.17E-10	5.98E-10	-6.57932	Down	S100 calcium binding protein A12
AQP9	-1.92229	1.86E-10	3.54E-10	-6.67337	Down	aquaporin 9
TREM1	-1.89896	1.62E-11	3.22E-11	-7.09344	Down	triggering receptor expressed on myeloid cells 1
BASP1	-1.84809	2.6E-12	5.37E-12	-7.39794	Down	brain abundant membrane attached signal protein 1
RGS18	-1.83021	6.46E-17	1.65E-16	-9.04689	Down	regulator of G protein signaling 18
TYROBP	-1.79113	3.8E-15	8.96E-15	-8.43274	Down	TYRO protein tyrosine kinase binding protein
BCL2A1	-1.78755	7.33E-13	1.55E-12	-7.60447	Down	BCL2 related protein A1
CSF3R	-1.7864	4.52E-11	8.84E-11	-6.91842	Down	colony stimulating factor 3 receptor
FCGR2A	-1.76976	6.33E-16	1.55E-15	-8.70563	Down	Fc fragment of IgG receptor IIa
FPR2	-1.76785	5.05E-11	9.85E-11	-6.89965	Down	formyl peptide receptor 2
LST1	-1.75056	6.17E-13	1.31E-12	-7.63239	Down	leukocyte specific transcript 1
UGT2B17	-1.73106	1.67E-09	3.05E-09	-6.28126	Down	UDP glucuronosyltransferase family 2 member B17
GNAT3	-1.7061	1.04E-16	2.63E-16	-8.97661	Down	G protein subunit alpha transducin 3
OR51E1	-1.69262	7.95E-11	1.54E-10	-6.82145	Down	olfactory receptor family 51 subfamily E member 1
MMP9	-1.68728	6.85E-10	1.27E-09	-6.44241	Down	matrix metallopeptidase 9
RIPOR2	-1.6709	3.33E-10	6.27E-10	-6.571	Down	RHO family interacting cell polarization regulator 2
CXCL8	-1.63914	4.6E-07	7.41E-07	-5.1916	Down	C-X-C motif chemokine ligand 8
PYGL	-1.63405	2.33E-17	6.06E-17	-9.19781	Down	glycogen phosphorylase L
CMYA5	-1.58592	2.01E-13	4.37E-13	-7.8125	Down	cardiomyopathy associated 5
PLA2G2A	-1.57032	3.53E-16	8.74E-16	-8.79354	Down	phospholipase A2 group IIA
KCNJ15	-1.56946	6.57E-12	1.33E-11	-7.24437	Down	potassium inwardly rectifying channel subfamily J member 15
ADGRE3	-1.54704	1.48E-14	3.38E-14	-8.22322	Down	adhesion G protein-coupled receptor E3

CXCR2	-1.54671	1.09E-11	2.19E-11	-7.16002	Down	C-X-C motif chemokine receptor 2
RGS13	-1.54387	2.18E-15	5.21E-15	-8.51774	Down	regulator of G protein signaling 13
BMX	-1.53703	2.83E-19	8.59E-19	-9.83734	Down	BMX non-receptor tyrosine kinase
HPGDS	-1.52735	9.32E-14	2.05E-13	-7.93473	Down	hematopoietic prostaglandin D synthase
PADI4	-1.52008	8.94E-11	1.73E-10	-6.80114	Down	peptidyl arginine deiminase 4
ALOX5AP	-1.5186	1.26E-24	5E-23	-11.5524	Down	arachidonate 5-lipoxygenase activating protein
FCN1	-1.50189	1.44E-09	2.63E-09	-6.30864	Down	ficolin 1
GABRP	-1.50022	4.13E-18	1.13E-17	-9.45081	Down	gamma-aminobutyric acid type A receptor pi subunit
PSTPIP2	-1.48225	5.17E-15	1.21E-14	-8.38564	Down	proline-serine-threonine phosphatase interacting protein 2
FCAR	-1.47755	2.65E-10	5.02E-10	-6.61101	Down	Fc fragment of IgA receptor
S100P	-1.4758	5.41E-19	1.59E-18	-9.74463	Down	S100 calcium binding protein P
LILRA2	-1.46484	2.39E-12	4.94E-12	-7.41189	Down	leukocyte immunoglobulin like receptor A2
DEFA6	-1.4532	3.85E-21	1.29E-20	-10.446	Down	defensin alpha 6
SH2D6	-1.45243	8.19E-10	1.51E-09	-6.41037	Down	SH2 domain containing 6
PROK2	-1.44931	2.68E-10	5.07E-10	-6.60902	Down	prokineticin 2
HCK	-1.43882	2.89E-13	6.25E-13	-7.75431	Down	HCK proto-oncogene, Src family tyrosine kinase
GCG	-1.42497	2.43E-11	4.82E-11	-7.0241	Down	glucagon
FYB1	-1.4225	2.31E-15	5.5E-15	-8.50909	Down	FYN binding protein 1
VNN2	-1.41657	1.04E-09	1.92E-09	-6.36652	Down	vanin 2
DCLK2	-1.40933	6.2E-10	1.15E-09	-6.46023	Down	doublecortin like kinase 2
GAPT	-1.38273	1.05E-12	2.21E-12	-7.546	Down	GRB2 binding adaptor protein, transmembrane
C11orf53	-1.38203	6.37E-16	1.56E-15	-8.70463	Down	chromosome 11 open reading frame 53
LRMP	-1.37944	4E-13	8.58E-13	-7.70226	Down	lymphoid restricted membrane protein
SRGN	-1.35639	1.3E-13	2.85E-13	-7.88154	Down	serglycin
LINC01659	-1.33744	2.84E-14	6.41E-14	-8.12165	Down	long intergenic non-protein coding RNA 1659
SMIM25	-1.33409	2.46E-10	4.66E-10	-6.62428	Down	small integral membrane protein 25
IGFBP6	-1.32576	9.92E-11	1.91E-10	-6.78301	Down	insulin like growth factor binding protein 6
LRRK2	-1.31787	2.32E-08	4E-08	-5.78906	Down	leucine rich repeat kinase 2
CHI3L1	-1.30335	3.22E-11	6.34E-11	-6.97635	Down	chitinase 3 like 1
PTGS1	-1.30256	1.29E-09	2.37E-09	-6.32805	Down	prostaglandin-endoperoxide synthase 1
GMFG	-1.29993	2.97E-13	6.41E-13	-7.7501	Down	glia maturation factor gamma
IFITM2	-1.29973	3.3E-12	6.79E-12	-7.3584	Down	interferon induced transmembrane protein 2
SIRPB1	-1.28055	3.73E-10	7.01E-10	-6.5508	Down	signal regulatory protein beta 1
ITGB2	-1.27683	1.21E-08	2.13E-08	-5.91262	Down	integrin subunit beta 2

LINC02381	-1.27637	4.36E-12	8.91E-12	-7.31258	Down	long intergenic non-protein coding RNA 2381
CMTM2	-1.27416	1.25E-08	2.19E-08	-5.9066	Down	CKLF like MARVEL transmembrane domain containing 2
TNFRSF10C	-1.27367	3.64E-12	7.47E-12	-7.34244	Down	TNF receptor superfamily member 10c
FCER1G	-1.27239	2.88E-12	5.94E-12	-7.38085	Down	Fc fragment of IgE receptor Ig
LOC105371267	-1.25916	4.82E-09	8.6E-09	-6.08622	Down	p53-regulated lncRNA 1
CD300LF	-1.24447	1.3E-10	2.49E-10	-6.73601	Down	CD300 molecule like family member f
S1PR4	-1.24308	2.16E-10	4.1E-10	-6.64734	Down	sphingosine-1-phosphate receptor 4
CFD	-1.23349	2.74E-12	5.65E-12	-7.38934	Down	complement factor D
REG3A	-1.23007	8.89E-13	1.88E-12	-7.57321	Down	regenerating family member 3 alpha
P2RY13	-1.229	2.7E-09	4.88E-09	-6.19313	Down	purinergic receptor P2Y13
ADAMTS18	-1.22052	3.48E-12	7.15E-12	-7.34974	Down	ADAM metallopeptidase with thrombospondin type 1 motif 18
GABRA4	-1.21806	1.23E-10	2.36E-10	-6.74605	Down	gamma-aminobutyric acid type A receptor alpha4 subunit
DCN	-1.21612	2.8E-08	4.82E-08	-5.75231	Down	decorin
DEFA5	-1.21483	1.82E-17	4.75E-17	-9.23425	Down	defensin alpha 5
HLA-DPB2	-1.21017	1.78E-12	3.7E-12	-7.46016	Down	major histocompatibility complex, class II, DP beta 2 (pseudogene)
ITLN2	-1.20882	1.69E-11	3.37E-11	-7.08585	Down	intelectin 2
MFAP5	-1.2029	2.16E-09	3.92E-09	-6.23427	Down	microfibril associated protein 5
ITPRID1	-1.20233	1.63E-08	2.83E-08	-5.85669	Down	ITPR interacting domain containing 1
GNG13	-1.20122	3.05E-09	5.5E-09	-6.17092	Down	G protein subunit gamma 13
HTR3E	-1.19469	3.99E-08	6.81E-08	-5.68372	Down	5-hydroxytryptamine receptor 3E
PRSS30P	-1.18774	3.74E-11	7.33E-11	-6.95102	Down	serine protease 30, pseudogene
KRT23	-1.18773	1.46E-13	3.2E-13	-7.86308	Down	keratin 23
GPC3	-1.18382	4.13E-11	8.09E-11	-6.93383	Down	glypican 3
DPYSL3	-1.17092	3.32E-10	6.26E-10	-6.57126	Down	dihydropyrimidinase like 3
FCGR3A	-1.16899	3.07E-08	5.27E-08	-5.73453	Down	Fc fragment of IgG receptor IIIa
TNFAIP6	-1.16808	4.51E-10	8.44E-10	-6.51716	Down	TNF alpha induced protein 6
IL17RB	-1.16514	6.19E-13	1.32E-12	-7.63188	Down	interleukin 17 receptor B
CSF2RA	-1.16435	4.46E-10	8.36E-10	-6.51891	Down	colony stimulating factor 2 receptor alpha subunit
DPEP2	-1.1561	1.51E-10	2.88E-10	-6.71012	Down	dipeptidase 2
GINS2	-1.15116	6.15E-15	1.43E-14	-8.35883	Down	GINS complex subunit 2
CEACAM4	-1.14827	1.13E-08	1.98E-08	-5.92678	Down	CEA cell adhesion molecule 4
NFE2	-1.14079	1.8E-09	3.28E-09	-6.2679	Down	nuclear factor, erythroid 2
AZGP1	-1.13437	1.66E-18	4.69E-18	-9.58281	Down	alpha-2-glycoprotein 1, zinc-binding
NCF2	-1.13264	5.84E-07	9.36E-07	-5.14168	Down	neutrophil cytosolic factor 2

AVIL	-1.13039	3.15E-11	6.2E-11	-6.98025	Down	advillin
CDA	-1.1102	8.79E-09	1.55E-08	-5.97363	Down	cytidinedeaminase
NTM	-1.10539	3.82E-12	7.84E-12	-7.33425	Down	neurotrimin
ITGAX	-1.10344	2.18E-08	3.77E-08	-5.80056	Down	integrin subunit alpha X
LINC01291	-1.09343	1.37E-07	2.27E-07	-5.43966	Down	long intergenic non-protein coding RNA 1291
TLR2	-1.09327	1.1E-09	2.02E-09	-6.35749	Down	toll like receptor 2
CSTA	-1.09275	7.07E-10	1.31E-09	-6.43677	Down	cystatin A
LILRB3	-1.08956	9.15E-14	2.02E-13	-7.93767	Down	leukocyte immunoglobulin like receptor B3
AZGP1P1	-1.08773	6.52E-11	1.27E-10	-6.85562	Down	AZGP1 pseudogene 1
ALOX5	-1.0861	2.38E-08	4.1E-08	-5.78412	Down	arachidonate 5-lipoxygenase
ADH1B	-1.08602	2.56E-10	4.83E-10	-6.61764	Down	alcohol dehydrogenase 1B (class I), beta polypeptide
GNLY	-1.08269	3.25E-07	5.29E-07	-5.26331	Down	granulysin
CTTNBP2	-1.07586	1.57E-10	3E-10	-6.70314	Down	cortactin binding protein 2
G0S2	-1.07514	8.21E-15	1.9E-14	-8.31443	Down	G0/G1 switch 2
PGLYRP1	-1.07458	3.04E-08	5.22E-08	-5.73655	Down	peptidoglycan recognition protein 1
S100A4	-1.07443	8.35E-17	2.12E-16	-9.00887	Down	S100 calcium binding protein A4
PTAFR	-1.07318	2.41E-08	4.16E-08	-5.78112	Down	platelet activating factor receptor
CREG2	-1.07196	1.89E-09	3.45E-09	-6.25845	Down	cellular repressor of E1A stimulated genes 2
CD14	-1.07177	1.2E-13	2.63E-13	-7.89459	Down	CD14 molecule
NRG2	-1.06712	1.39E-09	2.55E-09	-6.31437	Down	neuregulin 2
SH2D7	-1.06627	2.65E-08	4.56E-08	-5.76324	Down	SH2 domain containing 7
POU2F3	-1.06521	3.02E-09	5.44E-09	-6.17285	Down	POU class 2 homeobox 3
PSCA	-1.06453	2.77E-06	4.29E-06	-4.80706	Down	prostate stem cell antigen
CES1	-1.05685	5.39E-09	9.59E-09	-6.06549	Down	carboxylesterase 1
PRSS2	-1.05308	3.26E-12	6.71E-12	-7.36054	Down	serine protease 2
CAMP	-1.05177	1.07E-09	1.98E-09	-6.36151	Down	cathelicidin antimicrobial peptide
NFIX	-1.04764	2.74E-19	8.32E-19	-9.84211	Down	nuclear factor I X
BATF3	-1.04628	5.43E-06	8.26E-06	-4.65722	Down	basic leucine zipper ATF-like transcription factor 3
EVI2B	-1.04404	2.45E-08	4.23E-08	-5.77816	Down	ecotropic viral integration site 2B
SERPING1	-1.04274	6.42E-08	1.08E-07	-5.59034	Down	serpin family G member 1
LTF	-1.03602	1.81E-06	2.84E-06	-4.89975	Down	lactotransferrin
B4GALNT4	-1.02932	1.35E-07	2.24E-07	-5.44267	Down	beta-1,4-N-acetyl-galactosaminyltransferase 4
SEMA7A	-1.02701	2.61E-10	4.94E-10	-6.61374	Down	semaphorin 7A (John Milton Hagen blood group)
TCF4	-1.0253	2.15E-09	3.91E-09	-6.23481	Down	transcription factor 4

SELENOM	-1.02365	6.17E-16	1.51E-15	-8.70949	Down	selenoprotein M
FAT3	-1.02341	5.14E-06	7.84E-06	-4.6695	Down	FAT atypical cadherin 3
NEUROG3	-1.02085	4.9E-06	7.47E-06	-4.6805	Down	neurogenin 3
CHST15	-1.01907	2.67E-08	4.6E-08	-5.76159	Down	carbohydrate sulfotransferase 15
SPARCL1	-1.0183	4.56E-07	7.36E-07	-5.19313	Down	SPARC like 1
LOC643802	-1.01226	9.06E-09	1.6E-08	-5.96786	Down	u3 small nucleolarribonucleoprotein protein MPP10-like
DKK3	-1.00785	8.66E-06	1.3E-05	-4.55109	Down	dickkopf WNT signaling pathway inhibitor 3
LGALS1	-1.00235	5.84E-12	1.19E-11	-7.26391	Down	galectin 1
FGR	-0.99994	2.53E-07	4.13E-07	-5.31498	Down	FGR proto-oncogene, Src family tyrosine kinase
KCNN3	-0.9998	6.96E-09	1.23E-08	-6.01762	Down	potassium calcium-activated channel subfamily N member 3
TIMP1	-0.9988	1.05E-12	2.21E-12	-7.5463	Down	TIMP metallopeptidase inhibitor 1
EFEMP1	-0.99551	7.59E-07	1.21E-06	-5.08646	Down	EGF containing fibulin extracellular matrix protein 1
VNN3	-0.9936	4.97E-07	8.01E-07	-5.17509	Down	vanin 3
ASPG	-0.99342	1.99E-08	3.44E-08	-5.81849	Down	asparaginase
HTRA3	-0.99294	3.36E-07	5.46E-07	-5.25654	Down	HtrA serine peptidase 3
CLEC4D	-0.99156	1.11E-06	1.75E-06	-5.00596	Down	C-type lectin domain family 4 member D
HOXA3	-0.99129	1.15E-07	1.92E-07	-5.47391	Down	homeobox A3
STMN1	-0.98831	6.06E-12	1.23E-11	-7.25784	Down	stathmin 1
SMOC2	-0.98598	1.21E-09	2.23E-09	-6.33934	Down	SPARC related modular calcium binding 2
CREB5	-0.98564	2.14E-06	3.33E-06	-4.86383	Down	cAMP responsive element binding protein 5
MATK	-0.97994	6.44E-06	9.76E-06	-4.61869	Down	megakaryocyte-associated tyrosine kinase
PECAM1	-0.96693	6.44E-09	1.14E-08	-6.032	Down	platelet and endothelial cell adhesion molecule 1
MT1L	-0.96498	1.39E-09	2.55E-09	-6.31438	Down	metallothionein 1L, pseudogene
ISLR	-0.96392	2.2E-06	3.43E-06	-4.85758	Down	immunoglobulin superfamily containing leucine rich repeat
PTHLH	-0.96272	7.58E-09	1.34E-08	-6.00148	Down	parathyroid hormone like hormone
SAMSN1	-0.95931	3.32E-09	5.97E-09	-6.15537	Down	SAM domain, SH3 domain and nuclear localization signals 1
LY96	-0.95758	1.61E-07	2.66E-07	-5.40643	Down	lymphocyte antigen 96
TMEM71	-0.95733	1.12E-06	1.77E-06	-5.00384	Down	transmembrane protein 71
KLK13	-0.95617	3.06E-07	4.99E-07	-5.27549	Down	kallikrein related peptidase 13
GABRB1	-0.95244	1.21E-07	2.01E-07	-5.46453	Down	gamma-aminobutyric acid type A receptor beta1 subunit
LINC00528	-0.95159	1.99E-08	3.44E-08	-5.81858	Down	long intergenic non-protein coding RNA 528
NMU	-0.94715	2.74E-06	4.24E-06	-4.80965	Down	neuromedin U
ADGRG3	-0.94291	3.75E-06	5.75E-06	-4.74043	Down	adhesion G protein-coupled receptor G3
CLEC3B	-0.94233	1.71E-06	2.68E-06	-4.91267	Down	C-type lectin domain family 3 member B

CHAT	-0.94185	3.12E-07	5.09E-07	-5.27144	Down	choline O-acetyltransferase
SFRP2	-0.94159	2.04E-05	3E-05	-4.35094	Down	secreted frizzled related protein 2
HOXA-AS3	-0.94085	3.83E-07	6.21E-07	-5.22925	Down	HOXA cluster antisense RNA 3
LOC107984660	-0.93861	9.76E-08	1.63E-07	-5.5072	Down	uncharacterized LOC107984660
HMX2	-0.93439	4.58E-07	7.38E-07	-5.19255	Down	H6 family homeobox 2
RARRES2	-0.93382	5.29E-08	8.96E-08	-5.62856	Down	retinoic acid receptor responder 2
FGF2	-0.93132	3.91E-07	6.32E-07	-5.22542	Down	fibroblast growth factor 2
NRCAM	-0.92949	1.67E-08	2.91E-08	-5.85163	Down	neuronal cell adhesion molecule
IGSF6	-0.92697	1.85E-07	3.05E-07	-5.37856	Down	immunoglobulin superfamily member 6
RUNX2	-0.92564	6.06E-09	1.08E-08	-6.04335	Down	RUNX family transcription factor 2
CSF2RB	-0.92127	0.000165	0.00023	-3.83075	Down	colony stimulating factor 2 receptor beta common subunit
RBP1	-0.92063	2.69E-09	4.86E-09	-6.19406	Down	retinol binding protein 1
GJC3	-0.91737	9.73E-09	1.71E-08	-5.95447	Down	gap junction protein gamma 3
LAMA1	-0.91673	3.18E-11	6.27E-11	-6.97839	Down	laminin subunit alpha 1
CA9	-0.91529	4.17E-08	7.11E-08	-5.6751	Down	carbonic anhydrase 9
C5AR1	-0.91295	8.09E-13	1.71E-12	-7.58852	Down	complement C5a receptor 1
SUCNR1	-0.91247	6.15E-07	9.86E-07	-5.13067	Down	succinate receptor 1
PILRA	-0.91107	2.01E-09	3.66E-09	-6.24739	Down	paired immunoglobin like type 2 receptor alpha
ZNF280B	-0.90704	1.11E-07	1.84E-07	-5.48235	Down	zinc finger protein 280B
ALOX12B	-0.90342	1.18E-06	1.86E-06	-4.99292	Down	arachidonate 12-lipoxygenase, 12R type
VIM	-0.90338	2.07E-12	4.29E-12	-7.43545	Down	vimentin
GCA	-0.90123	4.11E-20	1.31E-19	-10.1124	Down	grancalcin
ARSL	-0.9002	8.12E-10	1.5E-09	-6.41182	Down	arylsulfatase L
NPY1R	-0.89988	7.22E-06	1.09E-05	-4.59281	Down	neuropeptide Y receptor Y1
PYY	-0.89785	6.46E-08	1.09E-07	-5.58913	Down	peptide YY
LUM	-0.8963	5.27E-06	8.03E-06	-4.66398	Down	lumican
PRDX4	-0.89295	5.53E-20	1.76E-19	-10.0702	Down	peroxiredoxin 4
GMPR	-0.89255	8.31E-08	1.4E-07	-5.53915	Down	guanosine monophosphate reductase
LINC00619	-0.88955	1.2E-05	1.79E-05	-4.47604	Down	long intergenic non-protein coding RNA 619
DIPK1C	-0.88949	2.42E-07	3.96E-07	-5.32405	Down	divergent protein kinase domain 1C
HOTAIRM1	-0.88797	2.75E-06	4.25E-06	-4.80915	Down	HOXA transcript antisense RNA, myeloid-specific 1
SLA	-0.88514	5.2E-09	9.27E-09	-6.07188	Down	Src like adaptor
QPCT	-0.87983	5.14E-08	8.73E-08	-5.63392	Down	glutaminyl-peptide cyclotransferase
FZD9	-0.87833	3.69E-07	5.98E-07	-5.23716	Down	frizzled class receptor 9

IRAK3	-0.87753	4.71E-06	7.19E-06	-4.68917	Down	interleukin 1 receptor associated kinase 3
H2AC20	-0.87742	8.13E-12	1.64E-11	-7.20881	Down	H2A clustered histone 20
LINC01558	-0.87093	4.65E-09	8.31E-09	-6.09273	Down	long intergenic non-protein coding RNA 1558
CLEC7A	-0.86899	8.29E-07	1.32E-06	-5.06784	Down	C-type lectin domain containing 7A
ARL3	-0.86846	1.15E-15	2.78E-15	-8.61519	Down	ADP ribosylation factor like GTPase 3
CPB1	-0.86599	7.73E-07	1.23E-06	-5.0825	Down	carboxypeptidase B1
NHP2	-0.86537	1.09E-17	2.89E-17	-9.30881	Down	NHP2 ribonucleoprotein
MGP	-0.86469	1.44E-06	2.27E-06	-4.94923	Down	matrix Gla protein
TFPI2	-0.8646	2.93E-06	4.53E-06	-4.79498	Down	tissue factor pathway inhibitor 2
PTCHD1	-0.86302	2.89E-06	4.46E-06	-4.79821	Down	patched domain containing 1
BEX3	-0.86286	3.25E-10	6.12E-10	-6.57508	Down	brain expressed X-linked 3
KLRB1	-0.86277	2.05E-09	3.72E-09	-6.24405	Down	killer cell lectin like receptor B1
TM4SF1	-0.86268	9.88E-06	1.48E-05	-4.52074	Down	transmembrane 4 L six family member 1
CENPH	-0.86213	3.92E-09	7.02E-09	-6.12471	Down	centromere protein H
CMSS1	-0.86072	1.55E-07	2.57E-07	-5.41408	Down	cms1 ribosomal small subunit homolog
PDZK1IP1	-0.86008	3.05E-08	5.23E-08	-5.73612	Down	PDZK1 interacting protein 1
C12orf77	-0.86002	7.8E-08	1.31E-07	-5.55183	Down	chromosome 12 open reading frame 77
ARHGDIB	-0.85952	2.39E-11	4.74E-11	-7.02687	Down	Rho GDP dissociation inhibitor beta
REG1A	-0.8595	1.03E-08	1.81E-08	-5.94381	Down	regenerating family member 1 alpha
PLG	-0.85344	2.14E-05	3.15E-05	-4.3394	Down	plasminogen
CLEC12A	-0.85252	5.81E-07	9.32E-07	-5.14264	Down	C-type lectin domain family 12 member A
BEX2	-0.85122	3.86E-10	7.24E-10	-6.54475	Down	brain expressed X-linked 2
AIF1	-0.8511	2.26E-09	4.1E-09	-6.226	Down	allograft inflammatory factor 1
GHRL	-0.85036	2.26E-05	3.31E-05	-4.32708	Down	ghrelin and obestatinprepropeptide
LOC441179	-0.84744	1.18E-09	2.17E-09	-6.34459	Down	uncharacterized LOC441179
SDC2	-0.84714	4.83E-08	8.21E-08	-5.64625	Down	syndecan 2
NCAM1	-0.84173	1.82E-05	2.69E-05	-4.37793	Down	neural cell adhesion molecule 1
SPI1	-0.83977	5.69E-06	8.64E-06	-4.6468	Down	Spi-1 proto-oncogene
TPH1	-0.83699	0.000243	0.000335	-3.72775	Down	tryptophan hydroxylase 1
ANXA1	-0.83667	8.09E-14	1.79E-13	-7.957	Down	annexin A1
MFAP4	-0.83639	4.44E-05	6.41E-05	-4.16334	Down	microfibril associated protein 4
DUT	-0.8362	1.84E-14	4.18E-14	-8.18954	Down	deoxyuridinetriphosphatase
ARG1	-0.83307	3.63E-05	5.27E-05	-4.21241	Down	arginase 1
UBE2C	-0.83225	1.09E-06	1.73E-06	-5.00957	Down	ubiquitin conjugating enzyme E2 C

ITGAM	-0.83087	1.15E-05	1.71E-05	-4.48649	Down	integrin subunit alpha M
KIT	-0.83048	2.36E-07	3.86E-07	-5.32937	Down	KIT proto-oncogene, receptor tyrosine kinase
SLCO4A1-AS1	-0.82969	3.2E-09	5.76E-09	-6.16193	Down	SLCO4A1 antisense RNA 1
PDCD2L	-0.82766	1.54E-08	2.68E-08	-5.86725	Down	programmed cell death 2 like
FAM241B	-0.82755	2.42E-07	3.96E-07	-5.32407	Down	family with sequence similarity 241 member B
KRT7	-0.82044	4.06E-06	6.22E-06	-4.72248	Down	keratin 7
GGH	-0.81874	3.66E-06	5.63E-06	-4.74544	Down	gamma-glutamyl hydrolase
SNORD3B-2	-0.81629	1.38E-05	2.05E-05	-4.44308	Down	small nucleolar RNA, C/D box 3B-2
RAD51AP1	-0.8148	1.54E-06	2.42E-06	-4.93524	Down	RAD51 associated protein 1
LINC02532	-0.81209	5.53E-07	8.88E-07	-5.15301	Down	long intergenic non-protein coding RNA 2532
SLC11A1	-0.80888	3.84E-07	6.22E-07	-5.2289	Down	solute carrier family 11 member 1
FEV	-0.80795	1.36E-05	2.02E-05	-4.44715	Down	FEV transcription factor, ETS family member
CFP	-0.8049	9.56E-08	1.6E-07	-5.51138	Down	complement factor properdin
SLC29A1	-0.80386	2.28E-06	3.54E-06	-4.85039	Down	solute carrier family 29 member 1 (Augustine blood group)
TAGLN3	-0.80338	5.86E-07	9.4E-07	-5.14074	Down	transgelin 3
LCN2	-0.80216	0.000336	0.000459	-3.6407	Down	lipocalin 2
C1R	-0.80124	1.88E-05	2.77E-05	-4.37086	Down	complement C1r
PTPRO	-0.80091	6.18E-05	8.84E-05	-4.08146	Down	protein tyrosine phosphatase receptor type O
RNF24	-0.79982	1.93E-15	4.61E-15	-8.5366	Down	ring finger protein 24
CORO2B	-0.79959	2.93E-07	4.78E-07	-5.28462	Down	coronin 2B
C1S	-0.7981	9.25E-05	0.000131	-3.97997	Down	complement C1s
NCCRP1	-0.7954	0.026208	0.031211	-2.23762	Down	NCCRP1, F-box associated domain containing
HMMR	-0.79432	2.04E-05	3E-05	-4.35137	Down	hyaluronan mediated motility receptor
RP9P	-0.79422	8.85E-11	1.71E-10	-6.80288	Down	RP9 pseudogene
PHACTR1	-0.79365	6.24E-09	1.11E-08	-6.03813	Down	phosphatase and actin regulator 1
PDCL3	-0.79108	3.1E-12	6.39E-12	-7.36861	Down	phosducin like 3
ALMS1P1	-0.79072	4.31E-07	6.96E-07	-5.20495	Down	ALMS1 pseudogene 1
EXOSC5	-0.78977	1.1E-09	2.02E-09	-6.35782	Down	exosome component 5
ADA2	-0.78854	7.31E-09	1.29E-08	-6.00841	Down	adenosine deaminase 2
DNAJC12	-0.78582	1.7E-06	2.66E-06	-4.91426	Down	DnaJ heat shock protein family (Hsp40) member C12
CDH26	-0.7857	4.47E-06	6.83E-06	-4.70098	Down	cadherin 26
CD200	-0.78548	6.73E-08	1.14E-07	-5.58097	Down	CD200 molecule
PTTG1	-0.78455	6.16E-06	9.34E-06	-4.62865	Down	PTTG1 regulator of sister chromatid separation, securin
LAGE3	-0.78419	3.22E-14	7.25E-14	-8.10186	Down	L antigen family member 3

DTD1	-0.78413	5.25E-12	1.07E-11	-7.28146	Down	D-aminoacyl-tRNAdeacylase 1
LYRM4	-0.78175	2.31E-09	4.18E-09	-6.22224	Down	LYR motif containing 4
GPX2	-0.78018	4.93E-09	8.8E-09	-6.0819	Down	glutathione peroxidase 2
FRMD6-AS1	-0.77992	4.81E-08	8.18E-08	-5.64699	Down	FRMD6 antisense RNA 1
CAV1	-0.77952	2.29E-06	3.55E-06	-4.84952	Down	caveolin 1
LILRB2	-0.77863	7.21E-06	1.09E-05	-4.59301	Down	leukocyte immunoglobulin like receptor B2
GSTA1	-0.7765	4.09E-16	1.01E-15	-8.77117	Down	glutathione S-transferase alpha 1
EPHB1	-0.77644	1.05E-06	1.67E-06	-5.01725	Down	EPH receptor B1
TCEA2	-0.77589	2.63E-09	4.75E-09	-6.19856	Down	transcription elongation factor A2
LGALS2	-0.7738	1.07E-13	2.35E-13	-7.91275	Down	galectin 2
MIR3142HG	-0.77228	1.32E-06	2.08E-06	-4.96814	Down	MIR3142 host gene
SNRNP27	-0.77092	5.05E-26	6.72E-24	-11.9879	Down	small nuclear ribonucleoprotein U4/U6.U5 subunit 27
APOBEC4	-0.77033	1.16E-07	1.93E-07	-5.47291	Down	apolipoprotein B mRNA editing enzyme catalytic polypeptide like 4
GRK5	-0.76969	6.8E-12	1.38E-11	-7.23845	Down	G protein-coupled receptor kinase 5
NHSL2	-0.7677	6.68E-07	1.07E-06	-5.11349	Down	NHS like 2
BIRC5	-0.76553	0.00011	0.000155	-3.9349	Down	baculoviral IAP repeat containing 5
ST8SIA6	-0.76454	9.63E-07	1.53E-06	-5.03593	Down	ST8 alpha-N-acetyl-neuraminide alpha-2,8-sialyltransferase 6
SLPI	-0.76269	5.64E-06	8.57E-06	-4.64884	Down	secretory leukocyte peptidase inhibitor
CPA3	-0.76184	5.82E-06	8.83E-06	-4.6417	Down	carboxypeptidase A3
APIP	-0.75836	8.49E-14	1.88E-13	-7.94941	Down	APAF1 interacting protein
GAU1	-0.75708	1.62E-06	2.53E-06	-4.92472	Down	GALNT8 antisense upstream 1
ASRGL1	-0.75703	1.75E-07	2.88E-07	-5.39035	Down	asparaginase and isoaspartyl peptidase 1
ARL9	-0.75687	5.33E-06	8.11E-06	-4.66162	Down	ADP ribosylation factor like GTPase 9
REG1B	-0.75584	0.004298	0.005437	-2.88419	Down	regenerating family member 1 beta
HTR3C	-0.75545	0.000857	0.00114	-3.37826	Down	5-hydroxytryptamine receptor 3C
PAFAH1B3	-0.7553	4.55E-13	9.74E-13	-7.68151	Down	platelet activating factor acetylhydrolase 1b catalytic subunit 3
OLFML2B	-0.75517	1.67E-05	2.47E-05	-4.39799	Down	olfactomedin like 2B
FFAR2	-0.75491	1.87E-09	3.4E-09	-6.2608	Down	free fatty acid receptor 2
SLC18A1	-0.75399	7.26E-05	0.000103	-4.0412	Down	solute carrier family 18 member A1
LOC101929653	-0.75121	8.43E-06	1.27E-05	-4.55726	Down	uncharacterized LOC101929653
ZRSR2	-0.74774	1.83E-22	6.72E-22	-10.8699	Down	zinc finger CCCH-type, RNA binding motif and serine/arginine rich 2
AJUBA	-0.74485	2.08E-05	3.06E-05	-4.34609	Down	ajuba LIM protein
TEX11	-0.74096	1.07E-06	1.69E-06	-5.01405	Down	testis expressed 11
LRMDA	-0.73822	3.27E-07	5.32E-07	-5.26189	Down	leucine rich melanocyte differentiation associated

MAP1B	-0.73787	2.14E-05	3.14E-05	-4.33984	Down	microtubule associated protein 1B
IQCM	-0.73734	8.98E-05	0.000127	-3.98753	Down	IQ motif containing M
TPD52L1	-0.73672	2.35E-09	4.25E-09	-6.21925	Down	TPD52 like 1
LOC105373383	-0.73665	1.35E-07	2.24E-07	-5.44249	Down	uncharacterized LOC105373383
NPL	-0.73414	1.07E-05	1.6E-05	-4.50171	Down	N-acetylneuraminate pyruvate lyase
PLEKHB1	-0.73395	8.44E-06	1.27E-05	-4.55711	Down	pleckstrin homology domain containing B1
PRRG3	-0.73243	1.04E-17	2.77E-17	-9.31547	Down	proline rich and Gla domain 3
ALPL	-0.72885	0.000942	0.001249	-3.35088	Down	alkaline phosphatase, biomineralization associated
GADD45GIP1	-0.72882	6.47E-23	2.49E-22	-11.0133	Down	GADD45G interacting protein 1
PBK	-0.7274	6.62E-05	9.45E-05	-4.06438	Down	PDZ binding kinase
LRRC25	-0.72682	3.82E-05	5.53E-05	-4.20028	Down	leucine rich repeat containing 25
RANBP1	-0.72626	3.42E-18	9.38E-18	-9.47813	Down	RAN binding protein 1
NOB1	-0.72543	2.76E-11	5.45E-11	-7.00288	Down	NIN1 (RPN12) binding protein 1 homolog
PCED1B-AS1	-0.72517	6E-06	9.1E-06	-4.63466	Down	PCED1B antisense RNA 1
PIK3CG	-0.72497	8.86E-06	1.33E-05	-4.54601	Down	phosphatidylinositol-4,5-bisphosphate 3-kinase catalytic subunit gamma
RNU4-1	-0.72444	8.02E-07	1.28E-06	-5.07475	Down	RNA, U4 small nuclear 1
REG4	-0.72421	8.83E-05	0.000125	-3.99174	Down	regenerating family member 4
MARC1	-0.72322	1.24E-10	2.39E-10	-6.74388	Down	mitochondrial amidoxime reducing component 1
IFI16	-0.72321	2.16E-10	4.09E-10	-6.64745	Down	interferon gamma inducible protein 16
ACOT12	-0.72317	4.85E-05	6.99E-05	-4.14148	Down	acyl-CoA thioesterase 12
ICAM3	-0.72272	3.25E-05	4.72E-05	-4.23988	Down	intercellular adhesion molecule 3
CD52	-0.71988	4.87E-07	7.84E-07	-5.17959	Down	CD52 molecule
SPTSSB	-0.71979	1.66E-05	2.46E-05	-4.39959	Down	serine palmitoyltransferase small subunit B
PRIM1	-0.71899	1.91E-09	3.47E-09	-6.25716	Down	DNA primase subunit 1
C19orf38	-0.71898	4.09E-07	6.62E-07	-5.21561	Down	chromosome 19 open reading frame 38
SIRPB2	-0.71858	9.81E-05	0.000139	-3.96503	Down	signal regulatory protein beta 2
SYCE1L	-0.71442	1.15E-06	1.82E-06	-4.99775	Down	synaptonemal complex central element protein 1 like
PNPLA4	-0.7134	6.64E-21	2.2E-20	-10.3694	Down	patatin like phospholipase domain containing 4
RPS4X	-0.71308	5.06E-21	1.69E-20	-10.4076	Down	ribosomal protein S4 X-linked
PART1	-0.71107	3.41E-05	4.95E-05	-4.22787	Down	prostate androgen-regulated transcript 1
PRKCQ-AS1	-0.71024	5.64E-08	9.55E-08	-5.61593	Down	PRKCQ antisense RNA 1
TANC2	-0.70923	8.4E-06	1.26E-05	-4.55818	Down	tetratricopeptide repeat, ankyrin repeat and coiled-coil containing 2
CCL25	-0.70859	1.36E-13	2.98E-13	-7.87433	Down	C-C motif chemokine ligand 25
LYAR	-0.70856	1.44E-10	2.75E-10	-6.71829	Down	Ly1 antibody reactive

MAD2L1	-0.70846	1.1E-05	1.65E-05	-4.49566	Down	mitotic arrest deficient 2 like 1
OSM	-0.70532	0.003017	0.003857	-2.99805	Down	oncostatin M
STAMBPL1	-0.70522	2.01E-12	4.17E-12	-7.44036	Down	STAM binding protein like 1
IGFBP3	-0.70516	0.000374	0.000509	-3.61146	Down	insulin like growth factor binding protein 3
PLEK	-0.70442	0.000495	0.000669	-3.53384	Down	pleckstrin
VRK1	-0.70275	1.43E-09	2.61E-09	-6.30981	Down	VRK serine/threonine kinase 1
GCAT	-0.70242	1.17E-09	2.14E-09	-6.34659	Down	glycine C-acetyltransferase
ITLN1	-0.7002	0.002379	0.003066	-3.07254	Down	intelectin 1
EWSAT1	-0.69692	1.17E-05	1.75E-05	-4.48121	Down	Ewing sarcoma associated transcript 1
LOC105376731	-0.69547	3.39E-05	4.93E-05	-4.22896	Down	taste receptor cell protein 1-like
RRM2	-0.69529	0.000413	0.000561	-3.58419	Down	ribonucleotidereductase regulatory subunit M2
CYSLTR1	-0.69287	4.79E-07	7.72E-07	-5.18288	Down	cysteinyl leukotriene receptor 1
ASCL2	-0.69276	2.01E-06	3.13E-06	-4.8779	Down	achaete-scute family bHLH transcription factor 2
IGFBP7	-0.69267	0.000117	0.000165	-3.91992	Down	insulin like growth factor binding protein 7
CNTLN	-0.69228	1.83E-07	3.01E-07	-5.38083	Down	centlein
TFB2M	-0.69091	1.13E-12	2.38E-12	-7.53386	Down	transcription factor B2, mitochondrial
LOC105370969	-0.69005	3.83E-11	7.5E-11	-6.94703	Down	uncharacterized LOC105370969
BRCA2	-0.68944	4.98E-08	8.46E-08	-5.64023	Down	BRCA2 DNA repair associated
CCNB2	-0.68935	2.72E-06	4.21E-06	-4.8111	Down	cyclin B2
SMYD3	-0.68823	1.65E-09	3.01E-09	-6.28373	Down	SET and MYND domain containing 3
DEPDC1B	-0.688	3.44E-06	5.3E-06	-4.75938	Down	DEP domain containing 1B
PRNP	-0.68762	2.2E-10	4.17E-10	-6.64401	Down	prion protein
HMGB2	-0.68732	1.19E-14	2.72E-14	-8.25735	Down	high mobility group box 2
HSBP1L1	-0.68695	2.61E-17	6.8E-17	-9.18061	Down	heat shock factor binding protein 1 like 1
TFB1M	-0.68692	6.77E-17	1.73E-16	-9.03989	Down	transcription factor B1, mitochondrial
CLEC2B	-0.68681	2.42E-07	3.97E-07	-5.32366	Down	C-type lectin domain family 2 member B
CCDC112	-0.68659	6.03E-17	1.54E-16	-9.0571	Down	coiled-coil domain containing 112
MT1H	-0.68643	1.35E-06	2.12E-06	-4.96442	Down	metallothionein 1H
NDUFA4L2	-0.68599	8.91E-05	0.000126	-3.98953	Down	NDUFA4 mitochondrial complex associated like 2
GIMAP4	-0.68586	1.79E-05	2.65E-05	-4.3816	Down	GTPase, IMAP family member 4
TUBG2	-0.68581	7.88E-09	1.39E-08	-5.99417	Down	tubulin gamma 2
DUSP14	-0.68577	3.81E-06	5.85E-06	-4.73661	Down	dual specificity phosphatase 14
TCEA3	-0.68406	1.8E-13	3.91E-13	-7.83056	Down	transcription elongation factor A3
ITGB3BP	-0.68356	7.85E-07	1.25E-06	-5.07942	Down	integrin subunit beta 3 binding protein

CCNB1	-0.68321	4.6E-05	6.63E-05	-4.15469	Down	cyclin B1
SERP2	-0.68282	2.07E-06	3.22E-06	-4.87111	Down	stress associated endoplasmic reticulum protein family member 2
FBXO16	-0.68242	2.85E-06	4.41E-06	-4.8008	Down	F-box protein 16
UBE2T	-0.68202	8.25E-05	0.000117	-4.00896	Down	ubiquitin conjugating enzyme E2 T
TEX45	-0.6818	2.38E-05	3.49E-05	-4.31442	Down	testis expressed 45
GSTA2	-0.68156	3.31E-14	7.44E-14	-8.09767	Down	glutathione S-transferase alpha 2
TUBA1A	-0.68135	1.42E-07	2.36E-07	-5.4316	Down	tubulin alpha 1a
TFF3	-0.6801	3.45E-07	5.6E-07	-5.25105	Down	trefoil factor 3
LYSMD2	-0.67844	1.01E-07	1.69E-07	-5.50035	Down	LysM domain containing 2
CCDC115	-0.67679	1.13E-18	3.24E-18	-9.63795	Down	coiled-coil domain containing 115
TRAPPC6A	-0.67667	1.45E-14	3.33E-14	-8.22584	Down	trafficking protein particle complex 6A
COL27A1	-0.67665	0.001993	0.002581	-3.12729	Down	collagen type XXVII alpha 1 chain
MYEOV	-0.67635	9.68E-07	1.54E-06	-5.03481	Down	myeloma overexpressed
NBPF25P	-0.67608	2.7E-06	4.18E-06	-4.81272	Down	NBPF member 25, pseudogene
MTSS1	-0.6758	2.42E-06	3.75E-06	-4.83725	Down	MTSS I-BAR domain containing 1
HMGN5	-0.67369	1.09E-15	2.65E-15	-8.62274	Down	high mobility group nucleosome binding domain 5
MACROH2A2	-0.67298	3.02E-08	5.19E-08	-5.73755	Down	macroH2A.2 histone
EFCAB2	-0.67142	9.42E-05	0.000133	-3.97547	Down	EF-hand calcium binding domain 2
MRPL24	-0.67047	6.04E-16	1.48E-15	-8.71251	Down	mitochondrial ribosomal protein L24
B3GNTL1	-0.67007	1.95E-06	3.05E-06	-4.88373	Down	UDP-GlcNAc:betaGal beta-1,3-N-acetylglucosaminyltransferase like 1
SVBP	-0.66935	8E-13	1.69E-12	-7.59039	Down	small vasohibin binding protein
ZG16B	-0.66934	0.000136	0.000191	-3.88055	Down	zymogen granule protein 16B
NUF2	-0.66866	0.001596	0.002082	-3.19479	Down	NUF2 component of NDC80 kinetochore complex
TGM3	-0.66848	0.0019	0.002466	-3.1419	Down	transglutaminase 3
GAL	-0.66812	0.00051	0.000689	-3.52545	Down	galanin and GMAP prepropeptide
LINC01091	-0.66782	1.2E-06	1.89E-06	-4.98943	Down	long intergenic non-protein coding RNA 1091
IL27RA	-0.66616	8.49E-08	1.42E-07	-5.53502	Down	interleukin 27 receptor subunit alpha
NDUFA8	-0.66391	3.02E-21	1.02E-20	-10.48	Down	NADH:ubiquinoneoxidoreductase subunit A8
CXCR4	-0.66333	0.000196	0.000271	-3.78588	Down	C-X-C motif chemokine receptor 4
EFCAB11	-0.66283	4.8E-08	8.16E-08	-5.6475	Down	EF-hand calcium binding domain 11
MRPL11	-0.66144	3.76E-11	7.37E-11	-6.95006	Down	mitochondrial ribosomal protein L11
CNN2	-0.66051	5.86E-10	1.09E-09	-6.47039	Down	calponin 2
PADI2	-0.65931	0.000937	0.001242	-3.35254	Down	peptidyl arginine deiminase 2
RBP7	-0.65875	3.39E-05	4.92E-05	-4.22951	Down	retinol binding protein 7

TRHDE	-0.65869	6.31E-08	1.07E-07	-5.59382	Down	thyrotropin releasing hormone degrading enzyme
DYRK4	-0.65852	8.58E-08	1.44E-07	-5.53279	Down	dual specificity tyrosine phosphorylation regulated kinase 4
RSL1D1	-0.65767	1.49E-17	3.93E-17	-9.26281	Down	ribosomal L1 domain containing 1
PMS1	-0.65748	7.63E-12	1.54E-11	-7.21926	Down	PMS1 homolog 1, mismatch repair system component
SPINK2	-0.65624	0.000695	0.00093	-3.43859	Down	serine peptidase inhibitor, Kazal type 2
MLN	-0.65434	0.001154	0.001521	-3.29155	Down	motilin
HSPA4L	-0.65258	6.56E-08	1.11E-07	-5.58593	Down	heat shock protein family A (Hsp70) member 4 like
NINJ2	-0.65086	1.13E-06	1.79E-06	-5.00106	Down	ninjurin 2
NIFK	-0.65067	4.13E-11	8.08E-11	-6.93404	Down	nucleolar protein interacting with the FHA domain of MKI67
PAK3	-0.64986	0.000118	0.000165	-3.91861	Down	p21 (RAC1) activated kinase 3
CLEC17A	-0.64974	0.000166	0.000231	-3.8287	Down	C-type lectin domain containing 17A
HAP1	-0.64918	0.000302	0.000413	-3.67006	Down	huntingtin associated protein 1
LOC100240735	-0.64912	2.28E-05	3.35E-05	-4.32462	Down	uncharacterized LOC100240735
EBPL	-0.64865	1.94E-08	3.36E-08	-5.82299	Down	EBP like
TMEFF2	-0.64863	0.001038	0.001372	-3.32271	Down	transmembrane protein with EGF like and two follistatin like domains 2
C3AR1	-0.64742	3.7E-05	5.36E-05	-4.20806	Down	complement C3a receptor 1
CHSY1	-0.64724	1.29E-08	2.26E-08	-5.90066	Down	chondroitin sulfate synthase 1
CCDC122	-0.64674	3.06E-11	6.03E-11	-6.98523	Down	coiled-coil domain containing 122
CASC1	-0.64425	4.01E-05	5.8E-05	-4.18834	Down	cancer susceptibility 1
CRYBA2	-0.6431	0.000169	0.000235	-3.82502	Down	crystallin beta A2
FBL	-0.64216	3.85E-10	7.23E-10	-6.54519	Down	fibrillarin
PTPN14	-0.64175	1.86E-05	2.74E-05	-4.3735	Down	protein tyrosine phosphatase non-receptor type 14
YPEL1	-0.64165	5.73E-06	8.7E-06	-4.64534	Down	yippee like 1
MRPL12	-0.6412	1.27E-07	2.1E-07	-5.45525	Down	mitochondrial ribosomal protein L12
PFDN2	-0.64085	7.38E-20	2.34E-19	-10.0292	Down	prefoldin subunit 2
SELPLG	-0.64013	3.31E-05	4.81E-05	-4.23543	Down	selectin P ligand

Table S2. The enriched GO terms of the up and down regulated differentially expressed genes.

GO ID	CATEGORY	GO Name	P Value	FDR B&H	FDR B&Y	Bonferroni	Gene Count	Gene
				**Up regulated genes**				
GO:0120036	BP	plasma membrane bounded cell projection organization	3.96E-13	1.02E-09	9.35E-09	2.05E-09	85	DLG5,ERBB2,CDC42EP4,ACHE,TRPM2,PDZD7,GPRIN1,PLXNB2,ADCY6,CHRNB2,AMIGO1,TAOK2,FZD1,DACT1,TUBB3,IGSF9,FGFR3,PLCG1,PLD2,VAV2,PLXNA2,PLXNB1,RASAL1,ACAP3,ARAP1,SEMA6C,PLXNA3,FLNA,VLDLR,EPS8L2,MINK1,CAMSAP3,SHC1,DOCK10,DNAH1,AATK,NSMF,ZNF335,PLXNA4,PPP1R16B,STK36,ZSWIM8,MYH9,TBC1D9B,MYO7A,CDHR2,INPPL1,SPTBN1,SARM1,RAP1GAP2,PLPPR5,ITGA3,CUL7,PRAG1,DCTN1,ITGB4,PLE

							KHM1,MAPK8IP3,PTK7,NTNG2,ARHGEF7,WDR90,NOTCH1,RAB35,RAP1GAP,HDAC6,RAPGEF4,SMURF1,LAMA5,LAMB2,LAMC1,PIP5K1C,CIT,LIF,SEMA3D,LINGO1,DAW1,LRP4,ROBO1,SCRIB,AGRN,EPHA10,DMTN,EVI5L,SEZ6
GO:0022008	BP	neurogenesis	3.83E-12	4.96E-09	4.53E-08	1.98E-08	85	DLG5,ERBB2,ACHE,PDZD7,GPRIN1,PLXNB2,ADCY6,CHRNB2,AMIGO1,TAOK2,FZD1,DACT1,TUBB3,GRK2,SUN2,JAG1,DISP3,IGSF9,FGFR3,PLCG1,PLD2,PLXNA2,PLXNB1,RASAL1,ACAP3,SEMA6C,PLXNA3,FLNA,VLDLR,MINK1,WNT9B,CAMSAP3,SHC1,DOCK10,NRBP2,AATK,NSMF,ZNF335,PLXNA4,DCHS1,GABRR2,ZSWIM8,MYO7A,INPPL1,SPTBN1,SARM1,RAP1GAP2,PLPPR5,ITGA3,CUL7,PRAG1,ITGB4,MAPK8IP3,PTK7,IGSF10,NTNG2,ARHGEF7,MYRF,DAGLA,MED12,NOTCH1,NOTCH3,CDON,PCSK9,RAB35,TCF3,RAP1GAP,HDAC6,HDAC5,RAPGEF4,SMURF1,LAMB2,LAMC1,PIP5K1C,CIT,LDLR,LIF,SEMA3D,LINGO1,LRP4,ROBO1,SCRIB,AGRN,EPHA10,SEZ6
GO:0043005	CC	neuron projection	3.36E-08	3.89E-06	2.75E-05	2.18E-05	67	ACHE,TRPM2,PDZD7,GPRIN1,CHRM4,CHRNA2,ADCY6,CHRNB2,AMIGO1,TAOK2,FZD1,TUBB3,GRK2,IGSF9,PLCG1,ACAP3,UNC13B,MAPK8IP1,FLNA,EPS8L2,ANK1,POLG,MINK1,DOCK10,TRIM3,AATK,NSMF,CTNND1,GABRR2,MYO1D,MYO7A,SPTBN1,SARM1,CPLX2,SREBF2,RAP1GAP2,RNF40,ITGA3,DCTN1,TIAM2,MAPK8IP3,ITPR3,NTNG2,ARHGEF7,SV2B,KCNK2,CACNA1H,BSN,DMWD,DAGLA,CACNA1C,RAP1GAP,CAD,HDAC6,RAPGEF4,NUMA1,SMURF1,CASR,SYT7,OPRK1,LRP4,ROBO1,CCDC120,ELK1,AGRN,EPHA10,SEZ6
GO:0005794	CC	Golgi apparatus	2.58E-07	1.52E-05	1.07E-04	1.67E-04	65	LZTR1,MAN2A2,ACHE,WDR81,ZDHHC8,ABCA7,ADCY3,FASN,AP1B1,CLTC,CHPF2,FGFR3,PLD2,MARCHF4,ARAP1,COL1A1,UNC13B,HSPG2,FLNA,NDST1,INPP5E,MINK1,MYO18A,CPD,PPP6R1,TRIM3,PITPNM1,SEC24C,TRPM4,CLSTN3,GBF1,INPPL1,SREBF1,SREBF2,FYCO1,CUL7,SLC30A1,CCDC88B,MAPK8IP3,DENND4B,TMEM132A,SEC16A,KIFC3,B4GALNT3,BSN,MYRF,BAIAP3,POFUT2,NOTCH1,NOTCH3,PCSK9,NPC1,RAB35,ZDHHC11B,RAP1GAP,HDAC5,NUMA1,GRINA,ATP9A,CIT,LDLR,ATP8B2,MALRD1,ZDHHC11,AGRN
GO:0008144	MF	drug binding	1.57E-07	4.53E-05	3.38E-04	1.54E-04	73	CDK18,ERBB2,ABCA2,ACHE,PFKFB3,ABCA7,CHRM4,ADCY3,CHRNA2,ADCY6,CHRNB2,TAOK2,TYK2,FASN,GRK2,CLCN7,CIITA,PI4KA,MAP3K10,FGFR3,CARNS1,OPLAH,CDC42BPG,HYOU1,MINK1,MYO18A,DGKD,SKIV2L,ZAP70,DNAH1,NLRP6,MST1R,NRBP2,AATK,DDX3Y,MAPK4,SNRNP200,PRKY,TRPM4,STK36,MYH9,MYO1D,MYO7A,MYO7B,DGKQ,CUL9,SIK1,TOR4A,IP6K1,MAST2,PRAG1,PTK7,ITPKB,ITPR3,PASK,ATP13A3,KIFC3,NOS2,NLRC3,DNHD1,CAD,RPS6KA4,GPT,MAP3K14,OAS3,UPF1,ATP9A,PIP5K1C,CIT,ATP8B2,KIFC2,AGRN,EPHA10
GO:0032553	MF	ribonucleotide binding	1.05E-06	1.28E-04	9.53E-04	1.02E-03	75	CDK18,ERBB2,ABCA2,PDE4A,PFKFB3,ABCA7,ADCY3,ADCY6,TAOK2,TYK2,TUBB3,GRK2,CLCN7,CIITA,PI4KA,MAP3K10,FGFR3,CARNS1,OPLAH,CDC42BPG,HYOU1,MINK1,MYO18A,DGKD,IRGQ,SKIV2L,ZAP70,DNAH1,NLRP6,MST1R,NRBP2,AATK,DDX3Y,MAPK4,SNRNP200,PRKY,TRPM4,STK36,MYH9,NDOR1,MYO1D,MYO7A,MYO7B,DGKQ,CUL9,SIK1,TOR4A,IP6K1,MAST2,PRAG1,PTK7,ITPKB,PASK,GVINP1,ARHGEF7,IFI44L,ATP13A3,KIFC3,NOS2,NLRC3,DNHD1,RAB35,RAP1GAP,CAD,RPS6KA4,MAP3K14,RAPGEF4,OAS3,UPF1,ATP9A,PIP5K1C,CIT,ATP8B2,KIFC2,EPHA10
**Down regulated genes**							
GO:0001775	BP	cell activation	1.50E-24	5.78E-22	5.30E-21	8.09E-21	95	CDH26,LILRB2,PECAM1,LRRK2,CLEC7A,CFP,SIRPB1,CHI3L1,FZD9,S100A8,S100A9,TYROBP,S100A12,S100P,FCAR,FCER1G,FCGR2A,FCGR3A,FCGR3B,FCN1,PIK3CG,QPCT,AIF1,GCA,BATF3,FGR,PLEK,MMP9,MNDA,ALOX5,CNN2,CD200,IL27RA,SELL,SELPLG,ADGRG3,CD300LF,ANXA1,FPR1,PRDX4,FPR2,GMFG,SUCNR1,ARG1,SLC11A1,CLEC4D,SLPI,FYB1,PRNP,CXCL8,CXCR1,CXCR2,PRSS2,RIPOR2,SPI1,GAPT,PTAFR,SAMSN1,ITGAM,ITGAX,ADA2,ITGB2,CLEC12A,GGH,CFD,CLEC4E,CXCR4,ADGRE3,ADAMTS18,PYGL,C3AR1,C5AR1,KIT,SPINK5,LST1,TCF4,LILRB3,LILRA2,PGLYRP1,CAMP,CAV1,RUNX2,LCN2,LGALS1,GAL,CD14,TIMP1,TLR2,IRAG2,PAK3,CDA,LTF,TNFAIP6,PADI2,EPHB1

GO:0046903	BP	secretion	8.35E-23	2.25E-20	2.06E-19	4.49E-19	101	LILRB2,PECAM1,LRRK2,CHAT,CFP,SIRPB1,CHI3L1,S100A8,S100A9,TYROBP,S100A12,S100P,FCAR,FCER1G,FCGR2A,FCGR3B,FCN1,PIK3CG,SVBP,QPCT,AIF1,PLA2G2A,GCA,FGR,PLG,PLEK,MMP9,MNDA,ALOX5,CNN2,ALOX12B,IL17RB,CD200,IL27RA,SELL,ADGRG3,ANXA1,FPR1,PRDX4,FPR2,SELENOM,GMFG,BIRC5,SUCNR1,AQP9,ARG1,SLC11A1,IGFBP3,CLEC4D,SLC18A1,SRGN,SLPI,CXCR1,CXCR2,PRSS2,GHRL,NCAM1,GCG,ISLR,PTAFR,ITGAM,ITGAX,ADA2,ITGB2,PTGS1,CLEC12A,NMU,GGH,CFD,CLEC4E,IL1R2,ADGRE3,SERPING1,PYGL,C3AR1,C5AR1,KIT,CA9,LILRB3,LILRA2,NPY1R,RARRES2,PGLYRP1,HAP1,FFAR2,CAMP,CAV1,LCN2,GAL,OSM,CD14,TIMP1,TLR2,IRAG2,TREM1,CDA,CLEC3B,LTF,TNFAIP6,PADI2,HCK
GO:0030141	CC	secretory granule	1.09E-33	5.89E-31	4.05E-30	5.89E-31	82	LILRB2,PECAM1,CFP,SIRPB1,CHI3L1,S100A8,S100A9,TYROBP,S100A12,S100P,FCAR,FCER1G,FCGR2A,FCGR3B,FCN1,QPCT,PLA2G2A,GCA,FGR,PLG,MMP9,MNDA,ALOX5,CNN2,SELL,ADGRG3,FPR1,PRDX4,FPR2,GMFG,CPA3,ARG1,SLC11A1,IGFBP3,CLEC4D,SRGN,SLPI,GNAT3,KLK13,CXCR1,CXCR2,PRSS2,GHRL,SPINK2,NCF2,GCG,ISLR,PTAFR,ITGAM,ITGAX,ADA2,ITGB2,CLEC12A,GGH,DEFA5,CFD,BRCA2,ADGRE3,SERPING1,PYGL,C3AR1,C5AR1,KIT,SPINK5,LILRB3,RARRES2,PGLYRP1,CAMP,REG1A,CAV1,LCN2,TFF3,GAL,CD14,TIMP1,TLR2,IRAG2,CDA,CLEC3B,LTF,TNFAIP6,PADI2
GO:0099503	CC	secretory vesicle	3.83E-32	1.04E-29	7.14E-29	2.08E-29	88	CTTNBP2,LILRB2,PECAM1,LRRK2,CFP,SIRPB1,CHI3L1,S100A8,S100A9,TYROBP,S100A12,S100P,FCAR,FCER1G,FCGR2A,FCGR3B,FCN1,QPCT,PLA2G2A,GCA,FGR,PLG,MMP9,MNDA,ALOX5,CNN2,SELL,ADGRG3,FPR1,PRDX4,FPR2,GMFG,CPA3,ARG1,SLC11A1,IGFBP3,CLEC4D,SLC18A1,SRGN,SLPI,GNAT3,KLK13,CXCR1,CXCR2,PRSS2,GHRL,SPINK2,NCF2,GCG,ISLR,PTAFR,ITGAM,ITGAX,ADA2,ITGB2,CLEC12A,GGH,DEFA5,CFD,BRCA2,ADGRE3,SERPING1,PYGL,C3AR1,C5AR1,KIT,SPINK5,DPYSL3,LILRB3,NPY1R,RARRES2,PGLYRP1,HAP1,CAMP,REG1A,CAV1,LCN2,TFF3,GAL,CD14,TIMP1,TLR2,IRAG2,CDA,CLEC3B,LTF,TNFAIP6,PADI2
GO:0005102	MF	signaling receptor binding	7.35E-08	2.28E-05	1.69E-04	6.85E-05	70	CDH26,LILRB2,CLEC7A,HMGB2,LY96,HOXA3,S100A4,S100A8,S100A9,TYROBP,S100A12,S100P,EFEMP1,FCN1,PIK3CG,PDCL3,FGF2,MLN,FGR,PLG,MMP9,CCL25,PROK2,SELPLG,SFRP2,CD300LF,SEMA7A,PILRA,ANXA1,FPR1,FPR2,ICAM3,GMFG,NRG2,BEX3,IGFBP6,CLEC4D,GNAT3,FYB1,DKK3,PRNP,CXCL8,GABRB1,GHRL,MTSS1,LAMA1,PYY,GCG,ITGAX,ADA2,ITGB2,PTHLH,NMU,PTPN14,RARRES2,PGLYRP1,HAP1,CMTM2,DUT,PSCA,REG1A,CAV1,ITPRID1,CCNB1,GAL,OSM,TIMP1,TLR2,PADI2,HCK
GO:0060089	MF	molecular transducer activity	1.74E-05	1.62E-03	1.20E-02	1.62E-02	63	IGSF6,REG4,LILRB2,CLEC7A,LY96,FZD9,OR51E1,EFEMP1,FCER1G,FCGR3A,FCN1,IL17RB,IL27RA,ADGRG3,FPR1,FPR2,NRG2,SUCNR1,CSF2RA,CSF2RB,CSF3R,DKK3,PRNP,CXCR1,S1PR4,CXCR2,GABRA4,GABRB1,GABRP,CYSLTR1,HTR3E,P2RY13,HTR3C,TNFRSF10C,PTAFR,ITGAX,ITGB2,CLEC4E,PTPRO,IL1R2,CXCR4,ADGRE3,C3AR1,C5AR1,KIT,KLRB1,LILRB3,LILRA2,NPY1R,PGLYRP1,FFAR2,DUT,PSCA,REG1A,REG1B,CCNB1,GAL,CD14,CCNB2,TLR2,TREM1,REG3A,EPHB1

Table S3. The enriched pathway terms of the up and down regulated differentially expressed genes.

Pathway ID	Pathway Name	P-value	FDR B&H	FDR B&Y	Bonferroni	Gene Count	Gene
					**Up regulated genes**		
1270303	Axon guidance	9.77E-04	1.16E-01	8.42E-01	7.99E-01	25	ERBB2,KL,CLTC,FGFR3,PLCG1,VAV2,PLXNA2,PLXNB1,RASAL1,PLXNA3,ANK1,SHC1,PLXNA4,MYH9,SPTBN1,ARHGEF7,CACNA1H,CACNA1C,RAP1GAP,RPS6KA4,LAMC1,PIP5K1C,ROBO1,AGRN,EPHA10
1270244	Extracellular matrix organization	3.71E-03	1.56E-01	1.00E+00	1.00E+00	15	PLEC,COL1A1,HSPG2,COL17A1,ADAMTS14,CAPN15,ADAM15,ITGA3,ITGB4,LAMA5,LAMB2,LAMC1,COL18A1,LRP4,AGRN

1269443	Signalling by NGF	3.81E-03	1.56E-01	1.00E+00	1.00E+00	21	PIK3AP1,ERBB2,ADCY3,ADCY6,KL,GRK2,CLTC,FGFR3,PLCG1,VAV2,RASAL1,SHC1,SPTBN1,TIAM2,ITPR3,ARHGEF7,RALGDS,ARHGEF18,PIP5K1C,LINGO1,ELK1
1270302	Developmental Biology	1.47E-02	3.01E-01	1.00E+00	1.00E+00	36	ERBB2,EVPL,KL,CLTC,FGFR3,PLCG1,VAV2,PLXNA2,PLXNB1,RASAL1,PLXNA3,ANK1,SHC1,PPL,ZNF335,PLXNA4,MYH9,SPTBN1,SREBF1,SREBF2,ARHGEF7,CACNA1H,MED12,NOTCH1,CDON,CACNA1C,TCF3,RAP1GAP,RPS6KA4,LAMC1,PIP5K1C,PKP3,ROBO1,AGRN,EPAS1,EPHA10
1269171	Adaptive Immune System	3.98E-02	3.97E-01	1.00E+00	1.00E+00	27	PIK3AP1,ERBB2,FZR1,KL,AP1B1,CLTC,FBXO41,FGFR3,PLCG1,TRAF7,SHC1,ZAP70,PPL,RNF123,SEC24C,RAP1GAP2,CUL7,DCTN1,NCR3LG1,ITPR3,KLHL21,RAP1GAP,MAP3K14,RAPGEF4,SMURF1,PIP5K1C,KLHL25
1270001	Metabolism of lipids and lipoproteins	8.50E-02	4.43E-01	1.00E+00	1.00E+00	25	ACHE,ZDHHC8,FASN,LMF2,PI4KA,CLTC,OSBPL7,PLA2G4F,PLD2,VLDLR,INPP5E,PITPNM1,SEC24C,INPPL1,SREBF1,SREBF2,TIAM2,PNPLA6,ARNT2,MED12,PCSK9,NPC1,PIP5K1C,LDLR,AKR1B15
**Down Regulated**							
1457780	Neutrophil degranulation	3.42E-24	2.61E-21	1.88E-20	2.61E-21	58	LILRB2,PECAM1,CFP,SIRPB1,CHI3L1,S100A8,S100A9,TYROBP,S100A12,S100P,FCAR,FCER1G,FCGR2A,FCGR3B,FCN1,QPCT,GCA,FGR,MMP9,MNDA,ALOX5,CNN2,SELL,ADGRG3,FPR1,PRDX4,FPR2,GMFG,ARG1,SLC11A1,CLEC4D,SLPI,CXCR1,CXCR2,PRSS2,PTAFR,ITGAM,ITGAX,ADA2,ITGB2,CLEC12A,GGH,CFD,ADGRE3,PYGL,C3AR1,C5AR1,LILRB3,PGLYRP1,CAMP,LCN2,CD14,TLR2,IRAG2,CDA,LTF,TNFAIP6,PADI2
1269203	Innate Immune System	1.09E-16	4.15E-14	2.99E-13	8.30E-14	83	LILRB2,PECAM1,CLEC7A,CFP,SIRPB1,LY96,CHI3L1,S100A8,S100A9,TYROBP,S100A12,S100P,FCAR,FCER1G,FCGR2A,FCGR3A,FCGR3B,FCN1,QPCT,FGF2,PLA2G2A,GCA,FGR,MMP9,MNDA,ALOX5,CNN2,SELL,ADGRG3,ITLN1,FPR1,PRDX4,FPR2,ICAM3,GMFG,NRG2,GNLY,IFI16,ARG1,SLC11A1,CSF2RA,CSF2RB,CLEC4D,SLPI,CXCR1,CXCR2,PRSS2,NCAM1,NCF2,PTAFR,ITGAM,ITGAX,ADA2,ITGB2,CLEC12A,GGH,DEFA5,DEFA6,CFD,CLEC4E,ADGRE3,C1R,PYGL,C1S,C3AR1,C5AR1,KIT,LILRB3,PGLYRP1,CAMP,LCN2,CD14,TLR2,IRAG2,TREM1,PAK3,REG3A,IRAK3,CDA,LTF,TNFAIP6,PADI2,HCK
1269576	G alpha (i) signalling events	5.42E-07	1.03E-04	7.45E-04	4.13E-04	21	CCL25,ANXA1,FPR1,FPR2,SUCNR1,GNAT3,CXCL8,CXCR1,S1PR4,CXCR2,GNG13,PYY,P2RY13,NMU,CXCR4,C3AR1,C5AR1,NPY1R,RGS13,GAL,RGS18
1269544	GPCR ligand binding	6.89E-06	5.84E-04	4.21E-03	5.25E-03	28	FZD9,MLN,CCL25,PROK2,ANXA1,FPR1,FPR2,SUCNR1,CXCL8,CXCR1,S1PR4,CXCR2,GHRL,CYSLTR1,GNG13,PYY,P2RY13,GCG,PTAFR,PTHLH,NMU,CXCR4,ADGRE3,C3AR1,C5AR1,NPY1R,FFAR2,GAL
1270244	Extracellular matrix organization	4.44E-05	2.82E-03	2.04E-02	3.39E-02	20	PECAM1,MFAP4,EFEMP1,FGF2,PLG,MMP9,SDC2,COL27A1,ICAM3,PRSS2,LAMA1,NCAM1,DCN,ITGAM,ITGAX,ITGB2,ADAMTS18,MFAP5,TIMP1,LUM
1269318	Signaling by Interleukins	6.04E-04	3.54E-02	2.55E-01	4.60E-01	26	FGF2,MMP9,ALOX5,IL17RB,IL27RA,VIM,ANXA1,FPR1,NRG2,BIRC5,CSF2RA,CSF2RB,CSF3R,CXCL8,NCAM1,PTAFR,ITGAM,ITGAX,ITGB2,IL1R2,KIT,LCN2,OSM,TIMP1,IRAK3,HCK

Biological Process(BP), Cellular Component(CC) and Molecular Functions (MF)

Table S4. Topology table for up and down regulated genes.

Regulation	Node	Degree	Betweenness	Stress	Closeness
Up	MYH9	231	0.083106	11909200	0.348923
Up	FLNA	196	0.07999	10927852	0.35285
Up	DCTN1	168	0.080808	7748054	0.330668
Up	CLTC	158	0.071579	7687192	0.351161
Up	ERBB2	158	0.069347	7857692	0.327216
Up	NOTCH1	156	0.079494	9958146	0.326941
Up	MYO18A	130	0.014844	2676542	0.316892
Up	PML	126	0.056341	7891820	0.329407
Up	HDAC6	117	0.054915	5893316	0.336828

Up	SEC16A	111	0.041283	4516670	0.333734
Up	KIFC3	110	0.044258	5676608	0.304433
Up	PLCG1	94	0.037489	3302572	0.322599
Up	SHC1	89	0.033089	3994096	0.330415
Up	TNS2	78	0.028831	3193368	0.287479
Up	UPF1	75	0.029592	4141234	0.307346
Up	FOXJ2	72	0.025448	4281228	0.269837
Up	TMEM63B	69	0.024305	2243442	0.28183
Up	CNOT1	61	0.023067	2619024	0.302632
Up	FZR1	61	0.020887	2279814	0.288932
Up	MED12	58	0.019843	3279514	0.268161
Up	SNRNP200	57	0.01318	2905516	0.29604
Up	USP20	55	0.018518	1919176	0.285851
Up	SMURF1	52	0.016676	2642582	0.284034
Up	PRPF8	49	0.011924	1673988	0.29983
Up	TYK2	44	0.011401	1809828	0.284491
Up	USP19	43	0.013204	1406194	0.282527
Up	SPTBN1	43	0.008444	1263038	0.311883
Up	SREBF2	41	0.010766	1784174	0.284595
Up	HDAC5	41	0.009427	1773292	0.280124
Up	CDK18	40	0.011774	1620634	0.291751
Up	TCF3	39	0.008725	1051044	0.273386
Up	PPP6R1	37	0.011599	836102	0.281565
Up	DMWD	36	0.0128	977744	0.288396
Up	TUBB3	36	0.009917	915362	0.297285
Up	TMEM132A	36	0.012522	1682324	0.267093
Up	CTNND1	35	0.009078	2098898	0.288975
Up	SCRIB	35	0.008757	1548182	0.293248
Up	NUMA1	34	0.008577	1678390	0.287266
Up	PLEC	33	0.011149	1518000	0.316557
Up	FGFR3	31	0.009978	1219886	0.270419
Up	NUP205	31	0.0068	1678252	0.280832
Up	SREBF1	30	0.006751	1233780	0.264926
Up	FASN	29	0.005503	1528992	0.285012
Up	ARHGEF7	29	0.007283	803468	0.300294
Up	MAPKBP1	28	0.006804	792976	0.272275
Up	GCN1	28	0.006355	1152120	0.281524
Up	MAP3K14	28	0.006365	1324702	0.272581
Up	EPAS1	28	0.006954	1100636	0.269444
Up	FYCO1	27	0.006095	824362	0.266598
Up	RNF40	27	0.007483	659526	0.275752
Up	ADAM15	27	0.005219	828418	0.280387
Up	ITGB4	27	0.006611	705452	0.293536
Up	FOSL2	26	0.007185	777256	0.266562
Up	ZAP70	26	0.005213	501414	0.285746
Up	MPRIP	26	0.001993	320704	0.298564
Up	GRK2	26	0.007851	709210	0.282691
Up	MYBBP1A	26	0.005321	1182570	0.289966
Up	PPL	24	0.007829	695410	0.283598
Up	AP1B1	23	0.004664	1193012	0.282239
Up	AATK	23	0.007433	1092768	0.257315
Up	PPP1R16B	23	0.005479	1076064	0.26007
Up	CAD	22	0.003373	376692	0.290747
Up	PLXNA3	22	0.005321	624980	0.248402
Up	HYOU1	22	0.007487	741310	0.27802

Up	INPPL1	21	0.003854	505262	0.282075
Up	MAPK8IP1	21	0.005349	555498	0.280893
Up	DLG5	21	0.00362	516518	0.258685
Up	RAB35	20	0.005577	721214	0.273675
Up	PTK7	19	0.00563	1103892	0.245717
Up	DOCK2	19	0.002529	415176	0.264422
Up	ELK1	18	0.002785	560550	0.270099
Up	EDC4	18	0.003062	367576	0.290834
Up	SETD7	18	0.004202	685686	0.268421
Up	ATXN2L	18	0.0033	637198	0.266562
Up	ANKFY1	18	0.005338	524976	0.245702
Up	SEC24C	17	0.002351	311964	0.288717
Up	MST1R	17	0.004103	678652	0.268106
Up	MCM3AP	17	0.004115	392266	0.270739
Up	ITPR3	17	0.002946	297934	0.280508
Up	MYO15B	17	0.003585	667898	0.264368
Up	SUN2	17	0.002803	298466	0.25601
Up	PKP3	16	0.003962	352604	0.287479
Up	SPECC1L	16	0.001354	171032	0.283847
Up	MAP3K10	16	0.002519	345136	0.255539
Up	RALGDS	16	0.004934	1197180	0.244681
Up	CGN	16	5.78E-04	132720	0.271155
Up	CCDC120	16	0.002569	828776	0.221129
Up	CNOT3	15	8.79E-04	85556	0.245531
Up	ZBTB7B	15	0.002981	436352	0.271628
Up	NEURL4	15	0.003567	265844	0.263812
Up	HSPG2	15	0.003442	488570	0.265415
Up	VAV2	15	0.002718	322246	0.285977
Up	TFE3	15	0.004081	253496	0.276556
Up	HIC1	15	0.003485	698408	0.246013
Up	SARM1	15	0.005388	445592	0.233006
Up	SGSM2	14	0.005277	923390	0.23832
Up	PLXNB1	14	0.003827	333106	0.229484
Up	BRPF1	14	0.003446	483314	0.24425
Up	POLE	14	0.003112	503172	0.258444
Up	SLC25A22	14	0.002789	366390	0.249135
Up	PLEKHG1	14	0.001719	299162	0.249567
Up	OAS3	14	0.002889	226356	0.243684
Up	MAPK8IP3	14	0.00236	403108	0.232964
Up	PLD2	13	0.002083	245424	0.282383
Up	NOS2	13	0.002821	251020	0.261328
Up	PAN2	13	0.003808	270874	0.254518
Up	SKIV2L	13	0.002807	569296	0.233482
Up	NUP188	13	0.002312	441004	0.258806
Up	GBF1	13	0.002156	543330	0.23873
Up	CAMSAP3	13	0.002315	267954	0.258221
Up	TNKS1BP1	12	0.002095	248304	0.254885
Up	LDLR	12	0.002881	383272	0.226041
Up	CIITA	12	0.002631	530768	0.242437
Up	ABCA2	12	0.003394	284740	0.269201
Up	EDEM1	12	0.003795	608306	0.21604
Up	CIC	12	0.00224	342592	0.23468
Up	POLRMT	11	0.003106	399074	0.245329
Up	ARAP1	11	0.001812	252668	0.271818
Up	MAPK4	11	0.002827	513354	0.244189

Up	CLCN7	11	9.41E-04	102048	0.246621
Up	COL17A1	3	1.61E-04	21788	0.244928
Up	MYO1D	2	6.19E-05	17036	0.264692
Up	RAVER1	1	0	0	0.232337
Up	EVPL	1	0	0	0.220953
Up	ZC3H3	1	0	0	0.218016
Up	PCSK9	1	0	0	0.184375
Up	MAN2A2	1	0	0	0.246481
Up	PLEKHN1	1	0	0	0.242498
Up	NDOR1	1	0	0	0.242498
Up	CMTR1	1	0	0	0.242498
Up	CASR	1	0	0	0.260837
Up	PASK	1	0	0	0.219146
Up	ELF4	1	0	0	0.247801
Up	SHROOM3	1	0	0	0.258685
Up	NOTCH3	1	0	0	0.246403
Up	JAG1	1	0	0	0.246403
Up	FAAP100	1	0	0	0.22587
Up	SNX33	1	0	0	0.218998
Up	SH3RF3	1	0	0	0.218998
Up	ROBO1	1	0	0	0.229173
Up	PLEKHM1	1	0	0	0.216715
Up	EPS8L2	1	0	0	0.223301
Up	KLHL15	1	0	0	0.223301
Up	ARNT2	1	0	0	0.212265
Up	CAND2	1	0	0	0.19714
Up	COL1A1	1	0	0	0.211239
Up	USP9Y	1	0	0	0.248371
Up	ZBTB7C	1	0	0	0.209451
Up	NPC1	1	0	0	0.244496
Up	PLXNB2	1	0	0	0.211434
Up	NDST1	1	0	0	0.210803
Up	SBF1	1	0	0	0.204665
Up	MAST2	1	0	0	0.219691
Up	CC2D1B	1	0	0	0.233397
Up	PCDH1	1	0	0	0.212115
Up	LRP4	1	0	0	0.209068
Up	HR	1	0	0	0.218838
Down	TCF4	186	0.080443	7982798	0.320102
Down	VIM	146	0.062238	9673084	0.327078
Down	LRRK2	114	0.046449	5881270	0.333305
Down	IFI16	91	0.035934	2890936	0.294671
Down	CAV1	74	0.034501	3642486	0.323593
Down	CCNB1	67	0.028307	3723968	0.305943
Down	TUBA1A	54	0.017017	1928674	0.312334
Down	HAP1	54	0.019204	1861478	0.274661
Down	TEX11	50	0.016505	2365480	0.276872
Down	EXOSC5	50	0.015531	2259640	0.264314
Down	LYAR	49	0.015456	1491732	0.2875
Down	MAD2L1	46	0.012966	1529406	0.285096
Down	FBL	43	0.01081	2684978	0.28063
Down	TCEA2	42	0.015079	1397158	0.304385
Down	BRCA2	42	0.013417	1411010	0.306159
Down	HCK	38	0.009214	1581626	0.275225
Down	S100A4	38	0.011937	1334286	0.308713

Down	RPS4X	38	0.008171	1891866	0.28069
Down	ITGB3BP	36	0.011135	1173054	0.277702
Down	PFDN2	34	0.010954	1159404	0.287883
Down	PAFAH1B3	34	0.007115	1225492	0.276655
Down	CREB5	33	0.007423	1042674	0.263704
Down	NUF2	33	0.009332	1497642	0.273232
Down	ANXA1	32	0.009055	1258680	0.300828
Down	S100A8	32	0.007253	969342	0.296288
Down	BEX2	32	0.006112	1138900	0.266342
Down	KIT	29	0.005841	995856	0.269332
Down	PTTG1	28	0.007971	885382	0.278778
Down	CD14	27	0.010174	1142284	0.252141
Down	MAP1B	26	0.007889	1000130	0.280792
Down	STAMBPL1	25	0.009389	938512	0.294849
Down	SPI1	25	0.007279	1669826	0.247864
Down	BIRC5	24	0.005049	829220	0.263062
Down	LGALS1	24	0.006761	794124	0.278438
Down	MRPL12	23	0.006117	474738	0.232074
Down	GADD45GIP1	23	0.007364	1019392	0.26103
Down	RUNX2	23	0.004951	787346	0.273232
Down	HMMR	22	0.006724	667876	0.283702
Down	MRPL11	22	0.005876	985534	0.25752
Down	CTTNBP2	21	0.004426	787798	0.24425
Down	CXCR4	19	0.005717	543656	0.273906
Down	S100A9	19	0.003264	447986	0.290096
Down	APIP	19	0.005534	501570	0.25672
Down	CHST15	19	0.007661	688762	0.242619
Down	NIFK	19	0.002299	238114	0.255892
Down	CLEC7A	18	0.006805	705330	0.230123
Down	ITGB2	18	0.005111	858702	0.255069
Down	CCNB2	17	0.003261	302214	0.284908
Down	CSF2RB	17	0.003237	336200	0.269556
Down	UBE2C	16	0.003855	338616	0.239716
Down	RSL1D1	16	0.002422	568444	0.266032
Down	BMX	16	0.003102	427964	0.270871
Down	BASP1	16	9.25E-04	116044	0.289254
Down	HMGB2	16	0.002726	287668	0.288717
Down	CENPH	16	0.002996	429184	0.232699
Down	PIK3CG	15	0.002579	343204	0.24376
Down	LAMA1	15	0.003191	501700	0.244789
Down	RRM2	15	0.002497	292850	0.266635
Down	ARL3	15	0.004764	699338	0.239583
Down	BEX3	15	0.002634	450090	0.22441
Down	NFIX	15	0.00342	599404	0.247848
Down	PBK	15	0.003952	470624	0.25672
Down	TLR2	14	0.006241	620072	0.256907
Down	SDC2	14	0.002352	187604	0.235077
Down	TPD52L1	14	0.003523	710758	0.244958
Down	TRAPPC6A	13	0.003875	385000	0.220589
Down	IGFBP3	13	0.003817	396058	0.267792
Down	ALOX5	13	0.004249	560968	0.229538
Down	NCF2	13	0.002568	384186	0.247202
Down	MRPL24	13	0.00178	265534	0.224799
Down	VRK1	13	0.003819	710302	0.268941
Down	NDUFA4L2	13	0.003033	413372	0.229308

Down	NHP2	13	0.002409	442338	0.256585
Down	HSPA4L	12	0.002179	357762	0.244373
Down	FGR	12	0.001217	240718	0.270871
Down	IRAK3	12	0.003972	375334	0.226067
Down	TNFAIP6	12	0.004525	497610	0.235804
Down	CXCL8	12	0.003383	302392	0.241789
Down	PTAFR	12	0.004579	353666	0.271401
Down	UBE2T	12	0.003223	341734	0.2313
Down	BATF3	12	0.002277	453320	0.2488
Down	MMP9	11	0.003658	450160	0.180556
Down	FGF2	11	0.002043	235084	0.227963
Down	STMN1	11	0.003185	307830	0.255842
Down	S100P	11	0.001764	315852	0.227323
Down	NDUFA8	11	0.002739	583540	0.24271
Down	PRDX4	11	0.001041	137274	0.238715
Down	BCL2A1	11	0.003206	376082	0.216161
Down	SMYD3	11	0.003453	535448	0.248006
Down	CXCR1	10	0.001934	164726	0.255186
Down	PLG	10	0.00265	266336	0.257964
Down	CXCR2	10	0.002068	216138	0.246621
Down	NFE2	10	0.001155	164280	0.229173
Down	CCDC112	10	0.001394	309290	0.234878
Down	AJUBA	10	0.00185	307250	0.233482
Down	DYRK4	10	0.002243	292244	0.24845
Down	PYGL	9	0.001357	199290	0.24209
Down	TIMP1	9	0.006389	867010	0.217418
Down	SLA	9	0.001338	164562	0.264584
Down	PECAM1	9	2.25E-04	38116	0.264548
Down	CCDC115	9	7.15E-04	107558	0.276222
Down	PTPN14	9	8.80E-04	142750	0.251212
Down	CMSS1	9	0.001802	478908	0.222559
Down	TNFRSF10C	8	0.001686	332028	0.218961
Down	FYB1	8	7.16E-04	168084	0.237374
Down	CSTA	8	0.001279	275406	0.237244
Down	PLEK	8	0.002115	303358	0.216667
Down	CSF3R	8	0.001004	108322	0.274719
Down	CDA	8	0.001648	243100	0.216269
Down	RANBP1	8	0.001213	204118	0.251228
Down	EFEMP1	8	0.001884	266664	0.234185
Down	PTPRO	8	0.001079	89316	0.239998
Down	LYSMD2	8	0.001347	151090	0.233538
Down	NTM	8	0.002058	193084	0.198499
Down	NOB1	8	0.001127	187878	0.246574
Down	TYROBP	7	1	20	1
Down	ARG1	7	8.37E-04	170482	0.247029
Down	GSTA1	7	0.001554	237926	0.209587
Down	CSF2RA	7	5.23E-04	40794	0.240845
Down	PRIM1	7	0.002087	229566	0.207661
Down	TFB1M	1	0	0	0.19701
Down	TREM1	1	0	0	0.555556
Down	LY96	1	0	0	0.204407
Down	PMS1	1	0	0	0.218016
Down	MATK	1	0	0	0.246559
Down	ITGAM	1	0	0	0.203242
Down	ITGAX	1	0	0	0.203242

Down	PLA2G2A	1	0	0	0.246481
Down	ALOX5AP	1	0	0	0.186695
Down	LCN2	1	0	0	0.152947
Down	PSTPIP2	1	0	0	0.248371
Down	DCLK2	1	0	0	0.248514
Down	NINJ2	1	0	0	0.187082
Down	SIRPB1	1	0	0	0.555556
Down	LTF	1	0	0	0.201378
Down	FPR1	1	0	0	0.220401
Down	PRNP	1	0	0	0.213478
Down	ADH1B	1	0	0	0.247801
Down	GCA	1	0	0	0.258685
Down	NMU	1	0	0	0.223301
Down	ALPL	1	0	0	0.214207

Table S5. miRNA - target gene and TF - target gene interaction.

Regulation	Target Genes	Degree	MicroRNA	Regulation	Target Genes	Degree	TF
Up	MYH9	116	hsa-mir-4329	Up	CLTC	59	SMARCA4
Up	ERBB2	73	hsa-mir-4315	Up	MYH9	53	TCF7
Up	MYO18A	71	hsa-mir-1299	Up	NOTCH1	45	MYB
Up	SEC16A	66	hsa-mir-4779	Up	SHC1	42	E2F4
Up	PLCG1	56	hsa-mir-3685	Up	KIFC3	42	CUX1
Up	NOTCH1	44	hsa-mir-623	Up	MYO18A	40	SALL4
Up	CLTC	31	hsa-mir-543	Up	PML	32	CTCF
Up	FLNA	23	hsa-mir-155-5p	Up	PLCG1	29	SOX17
Up	SHC1	10	hsa-mir-5582-5p	Up	ERBB2	24	MTF2
Up	KIFC3	8	hsa-mir-5193	Up	UPF1	23	ZFX
Up	HDAC6	5	hsa-mir-548m	Up	HDAC6	22	TFAP2A
Up	UPF1	5	hsa-mir-4635	Up	SEC16A	21	CEBPB
Up	DCTN1	4	hsa-mir-361-5p	Up	FLNA	18	SETDB1
Up	PML	3	hsa-mir-378a-3p	Up	DCTN1	15	RBPJ
Up	TNS2	2	hsa-mir-26b-5p	Down	TCF4	50	NANOG
Down	CCNB1	84	hsa-mir-6134	Down	VIM	43	GFI1B
Down	CAV1	58	hsa-mir-4459	Down	CAV1	36	GATA4
Down	VIM	30	hsa-mir-6124	Down	FBL	33	HIF1A
Down	HAP1	22	hsa-mir-9500	Down	TUBA1A	32	CLOCK
Down	MAD2L1	17	hsa-mir-1297	Down	EXOSC5	29	FLI1
Down	TCF4	15	hsa-mir-203a-3p	Down	MAD2L1	27	AP1S2
Down	BRCA2	12	hsa-mir-1245a	Down	TCEA2	26	MYCN
Down	TUBA1A	9	hsa-mir-744-5p	Down	BRCA2	26	E2F1
Down	IFI16	6	hsa-mir-136-5p	Down	LYAR	22	AR
Down	LRRK2	4	hsa-mir-582-3p	Down	IFI16	19	REST
Down	FBL	3	hsa-mir-181b-5p	Down	CCNB1	18	KLF4
Down	TCEA2	2	hsa-mir-17-5p	Down	LRRK2	16	SUZ12
Down	LYAR	1	hsa-mir-16-5p	Down	HAP1	15	RUNX1
Down	EXOSC5	1	hsa-mir-31-5p	Down	TEX11	6	SMAD4
